# Dual‐Targeted Nanovesicles Induced Cancer Stem‐Like Cell Differentiation to Sensitize Hepatocellular Carcinoma Radiotherapy

**DOI:** 10.1002/advs.202502409

**Published:** 2025-07-23

**Authors:** Hongmei Cao, Qian Wang, Yanan Niu, Shuxiang Wang, Haixue Jia, Dianyu Wang, Jinjian Liu, Wei Yuan, Lijun Yang, Jianfeng Liu

**Affiliations:** ^1^ State Key Laboratory of Advanced Medical Materials and Devices Tianjin Key Laboratory of Radiation Medicine and Molecular Nuclear Medicine Key Laboratory of Radiopharmacokinetics for Innovative Drugs Tianjin Institutes of Health Science Institute of Radiation Medicine Chinese Academy of Medical Sciences & Peking Union Medical College Tianjin 300192 P. R. China; ^2^ State Key Laboratory of Molecular Oncology National Cancer Center/National Clinical Research Center for Cancer/Cancer Hospital Chinese Academy of Medical Sciences and Peking Union Medical College Beijing 100021 P. R. China

**Keywords:** biomimetic nanovesicles, cancer stem‐like cells, differentiation therapy, dual‐targeted Pin1 and Notch1, radiosensitization

## Abstract

Highly plastic cancer stem‐like cells (CSCs) in hepatocellular carcinoma (HCC) drive tumor heterogeneity, contributing to radiotherapy failure. Although inducing CSC differentiation is proven effective in leukemia, this approach is shown limited success in solid tumors due to the complex signaling networks that sustain CSC stemness. In this study, the synergistic effect of Pin1 and Notch1 in HCC is identified, which plays a pivotal role in maintaining the aggressiveness of CSCs and promoting radioresistance. Building on this discovery, biomimetic nanovesicles (CALT‐GM‐NVs) are engineered by infusing tumor cell membranes into liposomes, which exhibit superior binding affinity to CSCs. RNA sequencing reveals that CALT‐GM‐NVs downregulate oncogenic signaling pathways while upregulating those linked to differentiation and apoptosis. In vivo, CALT‐GM‐NVs significantly reduced CSC‐driven radiotolerance and improved radiotherapy efficacy in both cell line‐derived and patient‐derived HCC xenograft models. These findings highlight the potential of simultaneously targeting Pin1 and Notch1 to induce CSC differentiation and provide a promising radiosensitizer for improving HCC radiotherapy outcomes.

## Graphical abstract

The existence of Pin1 and Notch1 crosstalk is identified and verified in HCC, and the crucial function of this crosstalk in sustaining the aggressive phenotypes of CSCs and radioresistance. A biomimetic nanovesicle is now developed to block the crosstalk between Pin1 and Notch1 for inducing CSCs differentiation and sensitizing HCC radiotherapy.

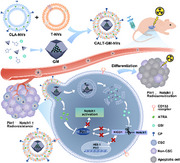

## 1 Introduction

Hepatocellular carcinoma (HCC) presents a significant clinical challenge due to its aggressive nature and high recurrence rates.^[^ ^1^ ^]^ Radiotherapy plays a vital role in managing HCC, particularly in controlling tumor size during early stages and providing palliative care in advanced cases, often complementing other treatments to achieve synergistic effects.^[^ ^2^ ^]^ However, the high heterogeneity of HCC often results in poor responses to radiotherapy.^[^ ^3^ ^]^ This heterogeneity is largely driven by cancer stem‐like cells (CSCs), which possess unique stem cell‐like properties that contribute to radioresistance.^[^ ^4^ ^]^ In HCC, CD133⁺ CSCs are known for their enhanced clonogenicity, proliferative capacity, and tumorigenicity, and are widely recognized as a representative CSC subset.^[^ ^5^ ^]^ Mechanistically, these CSC subsets activate key signaling pathways, such as Notch, Wnt/β‐catenin, and Hedgehog, which enhance their ability to self‐renew and repair DNA, further exacerbating their resistance to radiotherapy.^[^ ^6^ ^]^ Consequently, targeting and eliminating CSCs presents a promising strategy to improve the effectiveness of radiotherapy in HCC.

Over the past decade, differentiation therapy has emerged as a promising approach to eradicate CSCs by inducing their maturation into less tumorigenic cells. This strategy has been highly effective in treating acute promyelocytic leukemia.^[^ ^7^ ^]^ However, applying this approach to solid tumors like HCC presents considerable challenges due to the complex and heterogeneous nature of the tumor microenvironment. Although some preliminary studies have explored differentiation therapy in HCC by inducing CSC differentiation to curb tumor growth, results have been inconsistent, likely due to the intricate oncogenic signaling networks that sustain CSC stemness.^[^ ^8^ ^]^ Therefore, a deeper understanding of the key regulatory mechanisms maintaining CSC self‐renewal in HCC is critical to overcoming these challenges.

Prolyl isomerase Pin1 is a pivotal regulator within oncogenic signaling networks and has been implicated in the progression of various solid tumors, including HCC. By modulating the function of over 60 oncoproteins and inhibiting more than 30 tumor suppressors, Pin1 exerts broad oncogenic roles and promotes the self‐renewal and maintenance of CSCs across diverse tumor types.^[^ ^9^ ^]^ Notably, Pin1‐knockout mice remain resistant to tumorigenesis despite elevated oncogenic stimuli, highlighting its essential role in tumor development.^[^ ^10^ ^]^ Although Pin1 has been associated with HCC progression,^[^ ^11^ ^]^ its specific function in CSC regulation within the HCC context remains poorly defined. In parallel, the Notch1 signaling pathway, which is frequently dysregulated in HCC, has emerged as a key driver of CSC maintenance, therapy resistance, and impaired differentiation. This stemness‐promoting function of Notch1 has been well documented in glioblastoma, pancreatic, and breast cancers, suggesting a conserved mechanism across multiple malignancies.^[^ ^12^ ^]^ In breast cancer, a reciprocal regulatory loop between Pin1 and Notch1 has been identified, where Notch1 transcriptionally activates Pin1, and Pin1 stabilizes the Notch1 intracellular domain (NICD1) by preventing its degradation through the E3 ubiquitin ligase Fbxw7α.^[^ ^13^ ^]^ This mutual reinforcement amplifies Notch signaling and sustains CSC phenotypes. However, it remains largely unclear whether this regulatory interaction occurs in HCC or plays a role in CSC‐driven radioresistance. To further investigate this relationship, we analyzed data from The Cancer Genome Atlas (TCGA), which revealed a significant correlation between Pin1 and Notch1 expression in HCC tumors (R = 0.31, *p* < 0.001), with their co‐overexpression being associated with poorer overall survival (*p* = 0.001) (Figure S1, Supporting Information). These findings suggest that Pin1 and Notch1 may synergistically enhance CSC stemness and contribute to HCC progression, providing a potential therapeutic target for disrupting this oncogenic axis.

Based on these observations, we hypothesize that simultaneously targeting both Pin1 and Notch1 could disrupt their synergistic oncogenic effects, potentially induce CSC differentiation, and enhance the radiosensitivity of HCC tumors. However, effective delivery of the classical Pin1 inhibitor all‐trans retinoic acid (ATRA)^[^ ^14^ ^]^ and Notch1 suppressor GSI^[^ ^15^ ^]^ to CSCs remains a challenge due to the poor drug solubility and bioavailability. Metal‐organic frameworks (MOFs) offer a promising drug delivery platform due to their biocompatibility and tunable properties, but their application is often limited by immune system recognition and insufficient tissue targeting.^[^ ^16^ ^]^ Recent advancements in cell membrane‐coated nanoparticles have shown promising results in overcoming these limitations, thanks to their tissue‐specific targeting, reduced immunogenicity, and extended circulation time.^[^ ^17^ ^]^ While traditional liposomal systems have been widely used for targeted peptide modifications, they suffer from poor targeting precision and rapid clearance. To address these issues, combining liposomes with cell membranes to create engineered biomimetic vesicles has shown significant improvements in both targeting efficiency and therapeutic efficacy. Therefore, we propose the development of biomimetic nanovesicles by integrating tumor cell membranes with CSC‐targeted peptide liposomes. This approach aims to specifically deliver ATRA and GSI to CSCs, enhancing both the precision and efficacy of the treatment.

**Scheme 1:**
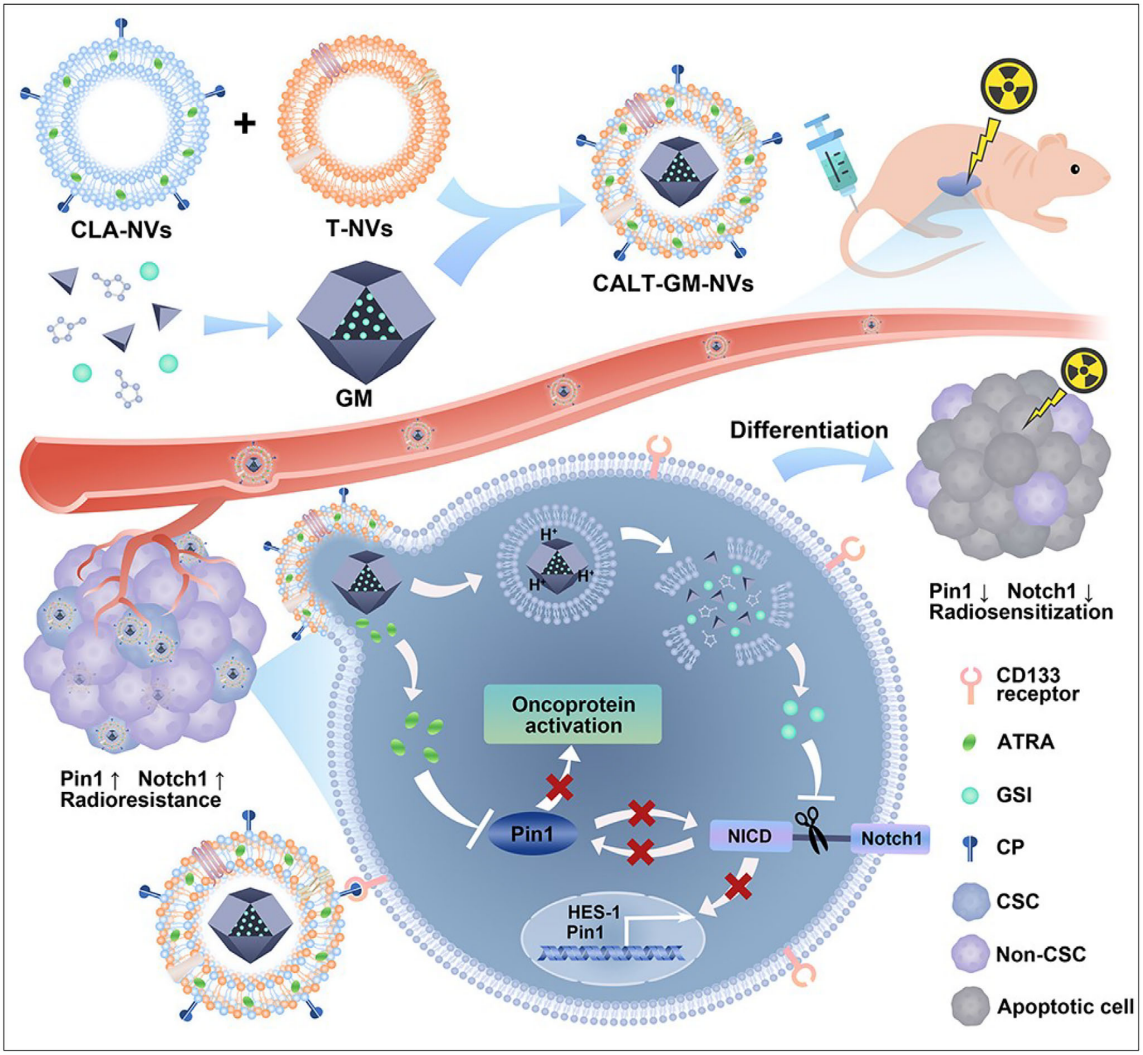
Schematic illustration of CALT‐GM‐NVs construction to destroy the Pin1 and Notch1 for CSC differentiation and HCC radiosensitization.

In this study, we explore the synergistic role of Pin1 and Notch1 in HCC, focusing on their regulation of CSC stemness and impact on radiotherapy resistance. We propose a combined inhibition strategy targeting both Pin1 and Notch1 to induce CSC differentiation and enhance HCC radiosensitivity. To achieve this, we engineer CSC‐targeted biomimetic nanovesicles by incorporating tumor cell membranes into peptide‐modified liposomes. These nanovesicles specifically target CSCs and release ATRA and GSI‐loaded MOFs (GM) through a membrane fusion process. After protonation of the imidazole group in acidic lysosomes,^[^ ^18^ ^]^ GM collapses, and the released GSI synergizes with ATRA in blocking multiple oncogenic signaling pathways, leading to increased apoptosis and differentiation of CSCs, while significantly reducing the proportion of CSCs and their colony‐forming ability (**Scheme** **1**). In animal experiments, these nanovesicles significantly improved the efficacy of radiotherapy, leading to prolonged survival in HCC‐bearing mice. These findings underscore the potential of our strategy to overcome CSC‐mediated resistance and improve radiotherapy outcomes for HCC, potentially leading to more effective treatments in clinical settings.

## 2 Results

### 2.1 Clinical Significance of Pin1 and Notch1 for HCC Radioresistance

**Figure 1:**
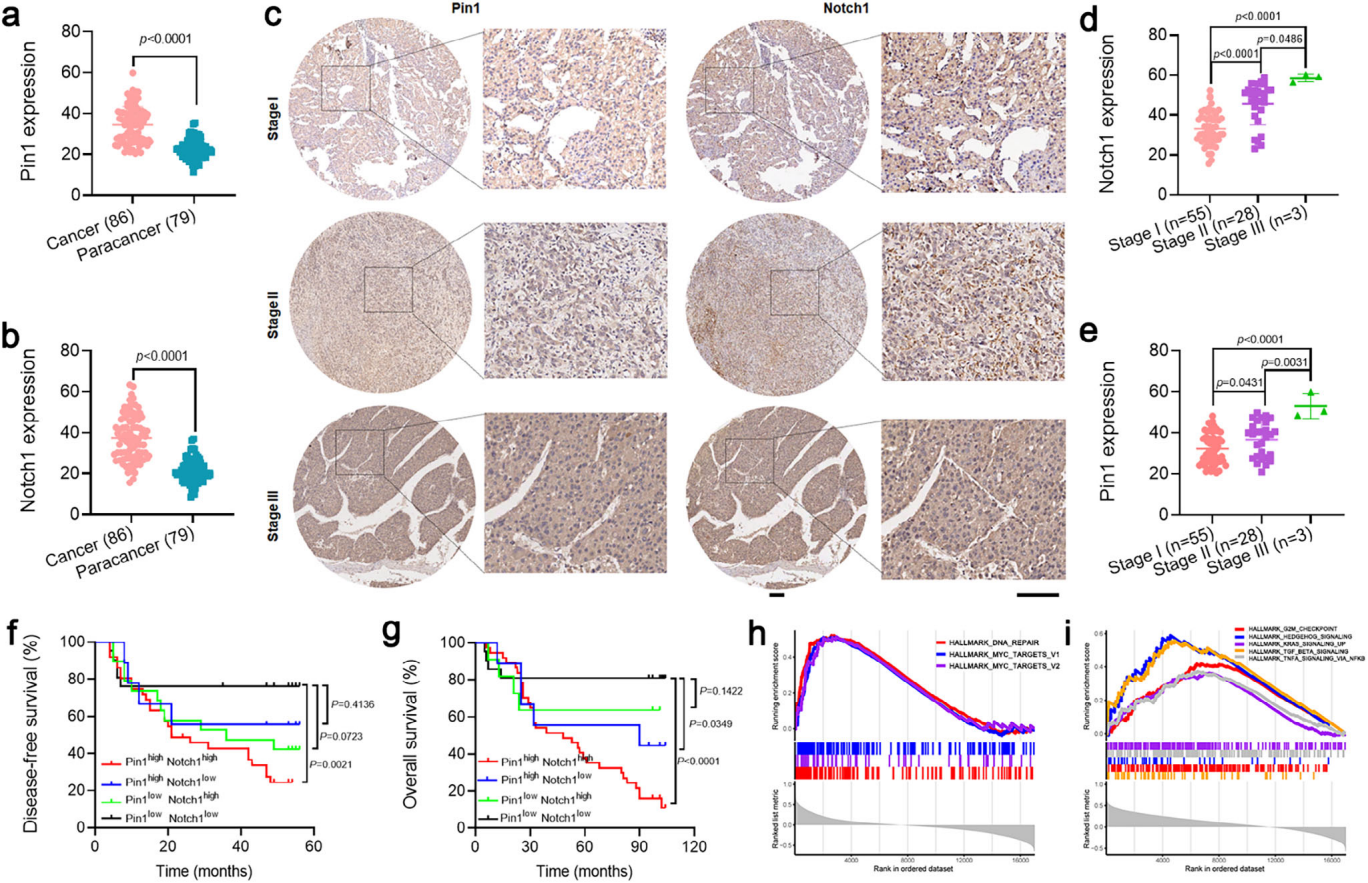
Clinical significance of Pin1 and Notch1 for HCC radioresistance. The Pin1 a) and Notch1 b) expression in tumor tissues (*n* = 86) and matched paratumor tissues (*n* = 79) from HCC patients. c) Representative immunohistochemical images of the Pin1 and Notch1 expression in tumors at different HCC stages. Quantitative analysis of the Pin1 d) and Notch1 e) expression in tumors at stage I (*n* = 56), II (*n* = 28), and III (*n* = 3). The disease‐free f) and overall g) survival of HCC patients with different Pin1 and Notch1 levels (*n* = 86). Gene set enrichment analysis (GSEA) of Pin1 h) and Notch1 i) expression related to intrinsic radioresistance‐related gene signatures in HCC patients. *p* Values were calculated by using one‐way ANOVA (a,b,d,e) and Log‐rank tests (f,g). Scale bars: 100 µm.

Given the observed strong correlation between Pin1 and Notch1 co‐expression and patient prognosis in HCC from the TCGA database (Figure S1, Supporting Information), we randomly selected a tissue microarray (TMA) comprising 86 tumor tissues and 79 matched paratumor tissues to further assess the expression of Pin1 and Notch1 in HCC and identify the correlation of their expression with HCC stages and patient survival. Following the findings from the TCGA database, we observed a substantial upregulation of Pin1 and Notch1 in HCC tissues compared with those in the neighboring normal liver tissues via immunohistochemical analysis (**Figure** **1**a,b; Figure S2a, Supporting Information). Both Pin1 and Notch1 levels were identified to be positively correlated with the HCC stages, with a progressive increase in their expression from stage I to stage II and then to stage III (Figure 1c–e; Figure S2b and Table S1, Supporting Information). Furthermore, though there were no apparent differences in the disease‐free survival between HCC patients with high Pin1 or Notch1 levels and those with low expression of both, the survival period was significantly shortened for patients with high levels of Pin1 and Notch1 (Figure 1f). Notably, the overall survival of HCC patients with upregulated Pin1 and Notch1 dramatically decreased to 16.2% within 90 months (*p* < 0.001), underscoring the vital role of Pin1 and Notch1 in the malignant progression of HCC (Figure 1g). Additionally, Gene set enrichment analysis (GSEA) for a gene set implicated in the Pin1 and Notch1 overexpression in HCC patients revealed that several intrinsic resistance pathways such as DNA repair, G2/M checkpoint, and KRAS signaling pathway associated with the maintenance of stemness features and poor prognosis in HCC were positively correlated with Pin1 and Notch1 (Figure 1h,i). These data demonstrated that the co‐expression of Pin1 and Notch1 is closely linked to the malignant progression and poor prognosis of HCC, highlighting their potential as key biomarkers and therapeutic targets.

### 2.2 Transcriptome Analysis Reveals Mechanisms of Pin1 and Notch 1 for CSCs Radioresistance

**Figure 2:**
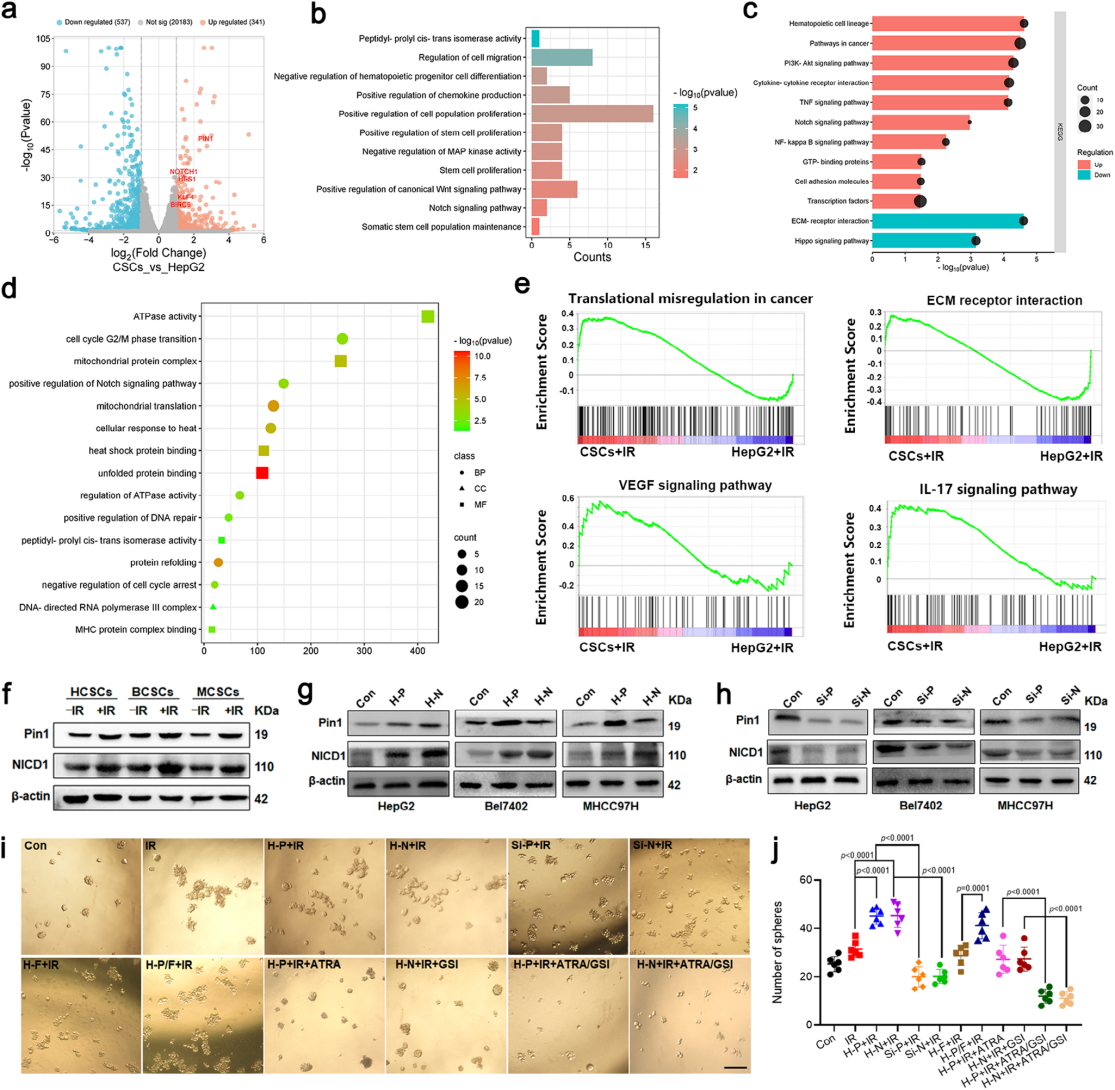
Transcriptome analysis reveals mechanisms of Pin1 and Notch 1 for CSCs radioresistance. a) Volcano plot of differentially expressed genes between HCSCs and HepG2 cells. b) GO pathway enrichment in HCSCs when compared to HepG2 cells. c) KEGG enrichment analysis in HCSCs. d) GO pathway enrichment in HCSCs+IR when compared to HCSCs. e) Enrichment analysis of GSEA gene set in HCSCs+IR. f) Immunoblotting of Pin1 and NICD1 in HCSCs, BCSCs, and MCSCs before and after 6 Gy of radiation. Immunoblotting analysis of Pin1, NICD1, and their negative regulator Fbxw7α in three HCC cell lines with either Pin1 or Notch1 overexpression (H‐P: Pin1‐high; H‐N: Notch1‐high) g) or Pin1 or Notch1 knockdown (Si‐P: siRNA‐Pin1; Si‐N: siRNA‐Notch1) h). Representative images i) and quantitative analysis j) of HCSC tumorspheres after the indicated treatments (H‐F: Fbxw7α‐high; H‐P/F: Pin1‐high and Fbxw7α‐high; ATRA/GSI: combination treatment with ATRA and GSI) (*n* = 6). Scale bar: 100 µm. *p* Values were calculated by using one‐way ANOVA.

Based on the high expression of Pin1 and Notch1 observed in clinical samples, and their significant correlation with HCC progression and poor patient prognosis, we sought to investigate the roles of Pin1 and Notch1 in maintaining CSC stemness and promoting radioresistance. To this end, we sorted the CD133^+^ population from HepG2, Bel7402, and MHCC97H cell lines. These sorted CD133^+^ cells (termed HCSCs, BCSCs, and MCSCs) tended to form tumorspheres in serum‐free cultures (Figure S3a,b, Supporting Information) and expressed high levels of stemness factors such as *Nanog*, *Oct4*, and *Sox2* (Figure S3c,d, Supporting Information), demonstrating the reliability of the CSC enrichment strategy. To elucidate the molecular basis of CSC‐mediated radioresistance, we performed transcriptomic profiling of HCSCs and HepG2 cells before and after irradiation. Principal component analysis (PCA) revealed the overall differences in gene expression between samples (Figure S4a, Supporting Information). Volcano plots and hierarchical clustering highlighted significant upregulation of *Pin1*, *Notch1*, and their downstream effectors (including *HES‐1* and *BIRC‐5*) were significantly upregulated in HCSCs (**Figure** **2**a; Figure S4b, Supporting Information). Furthermore, KEGG and GO enrichment analysis identified that pathways related to stem cell proliferation and maintenance, such as Notch and Wnt signaling, as well as oncogenic pathways including PI3K‐Akt, MAPK, and NF‐κB, were significantly upregulated in HCSCs (Figure 2b,c). Upon irradiation, CSCs but not HepG2 cells displayed increased Pin1 activity, Notch1 pathway activation, and enrichment of radioresistance‐related biological processes such as DNA repair and cell cycle regulation (Figure 2d; Figure S4c, Supporting Information). GSEA further revealed significantly elevated activity in pathways such as ECM–receptor interaction, VEGF, and IL‐17 signaling, all of which are closely associated with tumor progression and the development of radioresistance (Figure 2e). These transcriptomic differences underscored the activation of critical pathways involved in stemness maintenance and radioresistance in CSCs.

To validate the biological relevance of these transcriptomic alterations, we examined the effects of radiation on various CSC subtypes. Upon 6 Gy irradiation, HCSCs, BCSCs, and MCSCs exhibited significant increases in both Pin1 and NICD1 protein levels, as well as upregulation of Pin1 mRNA and Notch1 target gene HES‐1 (Figure 2f; Figure S5, Supporting Information). These changes were accompanied by elevated levels of core stemness‐associated transcription factors (Figure S6, Supporting Information), implying that radiation may enhance CSC self‐renewal in association with activation of the Pin1‐Notch1 axis. As expected, overexpression of either Pin1 or Notch1 significantly upregulated NICD1 and Pin1 protein levels (Figure 2g), whereas knockdown of either gene led to a marked reduction in NICD1 or Pin1 expression (Figure 2h). At the transcriptional level, activation of the Pin1‐Notch1 axis induced expression of canonical Notch targets (*HES‐1*, *HEY‐1*, *KLF‐4*, *BIRC‐5*), while knockdown of either gene reduced these targets (Figure S7, Supporting Information), highlighting their cooperative role in maintaining CSC signaling. Functionally, both Pin1 and Notch1 enhanced tumor sphere formation following irradiation, whereas their knockdown or overexpression of the negative regulator Fbxw7α significantly impaired CSC clonogenicity. Notably, the inhibitory effect of Fbxw7α was largely rescued by co‐overexpression of Pin1, suggesting that Pin1 counteracts Fbxw7α‐mediated NICD1 degradation, consistent with previous findings in breast CSCs.^[^ ^19^ ^]^ Moreover, the pro‐stemness effects induced by Pin1 and Notch1 overexpression were nearly abolished by combined treatment with ATRA and GSI, highlighting the therapeutic potential of dual pharmacological inhibition (Figure 2i,j). Quantitative analysis using the Chou–Talalay method^[^ ^20^ ^]^ yielded a combination index (CI₅₀) of 0.71 (Table S2, Supporting Information), indicating a synergistic interaction between ATRA and GSI. To further evaluate the clinical relevance of CSC‐mediated radioresistance, primary HCC cells were isolated and subjected to 6 Gy irradiation. Clonogenic assays revealed that irradiated cells displayed significantly enhanced colony‐forming capacity, consistent with selective CSC enrichment following radiation exposure (Figure S8, Supporting Information). Collectively, these findings highlight the central role of Pin1‐Notch1 crosstalk in regulating CSC‐driven radioresistance and suggest that dual targeting of this axis may offer a promising therapeutic approach for preventing HCC recurrence.

### 2.3 Construction and Characterization of CALT‐GM‐NVs

**Figure 3:**
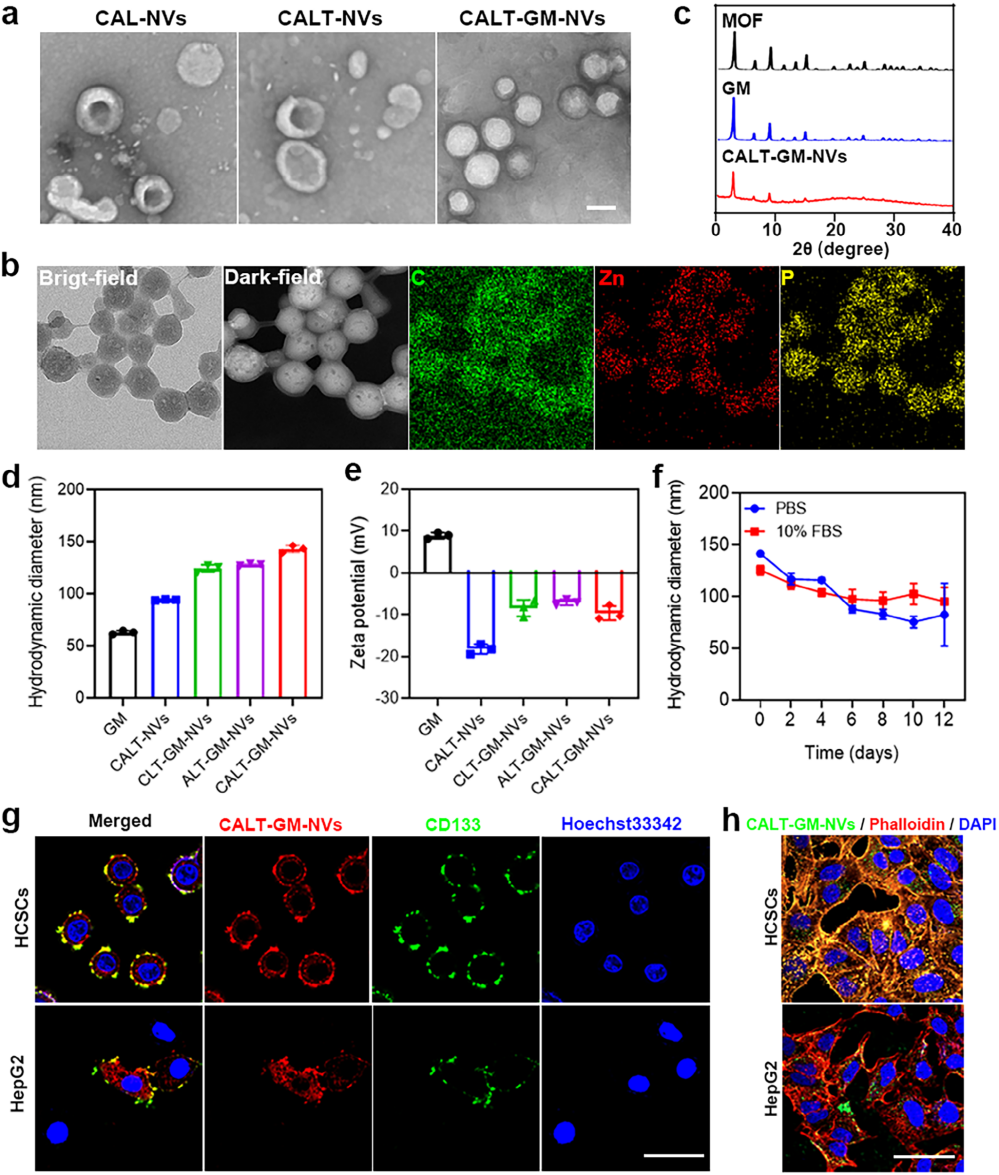
Construction and characterization of CALT‐GM‐NVs. a) Representative TEM images of CAL‐NVs, CALT‐NVs, and CALT‐GM‐NVs. Scale bar: 100 nm. b) Representative EDS mapping of CALT‐GM‐NVs. c) XRD patterns of MOFs, GM, and CALT‐GM‐NVs. Hydrodynamic diameters d) and zeta potentials e) of GM, CALT‐NVs, CLT‐GM‐NVs, ALT‐GM‐NVs, and CALT‐GM‐NVs (*n* = 3). f) The stability of CALT‐GM‐NVs in PBS with or without 10% of FBS over time (*n* = 3). g) Co‐localization of DiI‐labeled CALT‐GM‐NVs (red) and FITC‐labeled CD133 antibodies (green) in HCSCs and HepG2 cells (Hoechst33342: blue) Scale bar: 50 µm. h) Cellular uptake study of DiO‐labeled CALT‐GM‐NVs (green) in HCSCs and HepG2 cells (phalloidin: red; DAPI: blue). Scale bar: 50 µm.

Using the classical one‐pot co‐precipitation method, we synthesized GM with a polygonal shape of approximately 80 nm, closely resembling blank MOFs in both morphology and size (Figure S9, Supporting Information). To achieve CSC‐targeting, we synthesized the WRLRWHSPLK peptide (CP)^[^ ^21^ ^]^ and conjugated it to NH2‐PEG2000‐DSPE, forming CP‐PEG2000‐DSPE (Figure S10, Supporting Information). This conjugate self‐assembled with ATRA to produce CP‐modified, ATRA‐loaded liposome nanovesicles (CAL‐NVs). Upon fusion of CAL‐NVs with the tumor cell membrane, engineered biomimetic nanovesicles (CALT‐NVs) were formed, exhibiting cup‐shaped structures. The GM was then incorporated into the aqueous phase of CALT‐NVs, yielding homogeneous layered nanospheres (CALT‐GM‐NVs) (**Figure** **3**a). Energy dispersive spectroscopy (EDS) mapping confirmed the uniform distribution of Zn and P elements, demonstrating the successful integration of GM into CALT‐NVs (Figure 3b). The crystal structure of the MOFs remained largely intact after the incorporation of GSI and coating with CALT‐NVs, though the lipid bilayer partially obscured the X‐ray diffraction (XRD) signals (Figure 3c). We also prepared two control biomimetic materials, including nanovesicles without CSC‐targeting (ALT‐GM‐NVs) or without ATRA (CLT‐GM‐NVs), both with a similar micromorphology to CALT‐GM‐NVs (Figure S11a, Supporting Information). The hydrodynamic diameter of CALT‐GM‐NVs was measured to be ≈143.0 ± 3.1 nm, which is similar to that of ALT‐GM‐NVs (127.9 ± 1.0 nm) and CLT‐GM‐NVs (124.3 ± 4.1 nm), and larger than that of the component unit GM (62.8 ± 1.8 nm) and CALT‐NVs (94.2 ± 1.4 nm) (Figure 3d). Moreover, the polydispersity index of GM was greater than that of CLT‐GM‐NVs, ALT‐GM‐NVs, and CALT‐GM‐NVs, signifying the enhanced size uniformity and stability after the lipid membrane camouflage (Table S3, Supporting Information). After being clothed with negatively charged CALT‐NVs (−18.2 ± 1.1 mV), the zeta potential was dramatically decreased from 8.9 ± 0.6 mV of GM to −9.6 ± 0.7 mV of CALT‐GM‐NVs, which was conducive to the blood circulation of this drug delivery system (Figure 3e). SDS‐PAGE analysis showed that the protein profiles of nanovesicles retained the complete set of membrane proteins from cell membranes, confirming that membrane fusion did not alter the protein compositions (Figure S11b, Supporting Information).

To evaluate the stability, GM and CALT‐GM‐NVs were dispersed in phosphate‐buffered saline (PBS) with or without 10% fetal bovine serum (FBS). GM showed aggregation after 6 days, with particle size increasing over time (Figure S11c,d, Supporting Information). In contrast, CALT‐GM‐NVs exhibited consistent morphology and size over 10 days, demonstrating the enhanced structural stability conferred by the lipid membrane coating (Figure 3f). Next, drug loading content (DLC) analysis revealed that ATRA and GSI were loaded into CALT‐GM‐NVs at 6.7% and 5.8%, respectively, with corresponding drug loading efficiencies (DLE) of 42.4% and 41.0% (Table 4). Subsequently, we examined the release profiles of ATRA and GSI under physiological (pH 7.4) and acidic tumor conditions (pH 5.5). In the presence of Triton X‐100, ATRA was almost fully released within 12 h under both pH conditions, while GSI was rapidly released only at pH 5.5. Without Triton X‐100, both drugs exhibited slow release rates, with less than 60% cumulative release over 24 h (Figure S12a, Supporting Information). Then, to evaluate the CSC‐targeting and internalization efficiency, we labeled CALT‐GM‐NVs with DiI. As exhibited in Figure 3g and Figure S12b (Supporting Information), DiI‐labeled CALT‐GM‐NVs exhibited significant co‐localization with FITC‐labeled CD133 antibodies after incubation with HCSCs at 4 °C for 40 min. In contrast, no noticeable overlap between DiI and FITC was observed in HepG2 cells, indicating that CALT‐GM‐NVs specifically recognized and targeted CSCs. Furthermore, the uptake and accumulation of CALT‐GM‐NVs in HCSCs were substantially higher than in HepG2 cells after 2 h of incubation at 37 °C (Figure 3h). These results suggested that the biomimetic nanovesicles can stably maintain drugs during circulation but responsively unload the cargos upon reaching CSCs, acting as a CSC‐specific acid‐stimulated drug delivery system.

### 2.4 CALT‐GM‐NVs Induced CSC Differentiation by Synergistically Inhibiting Pin1 and Notch1

**Figure 4:**
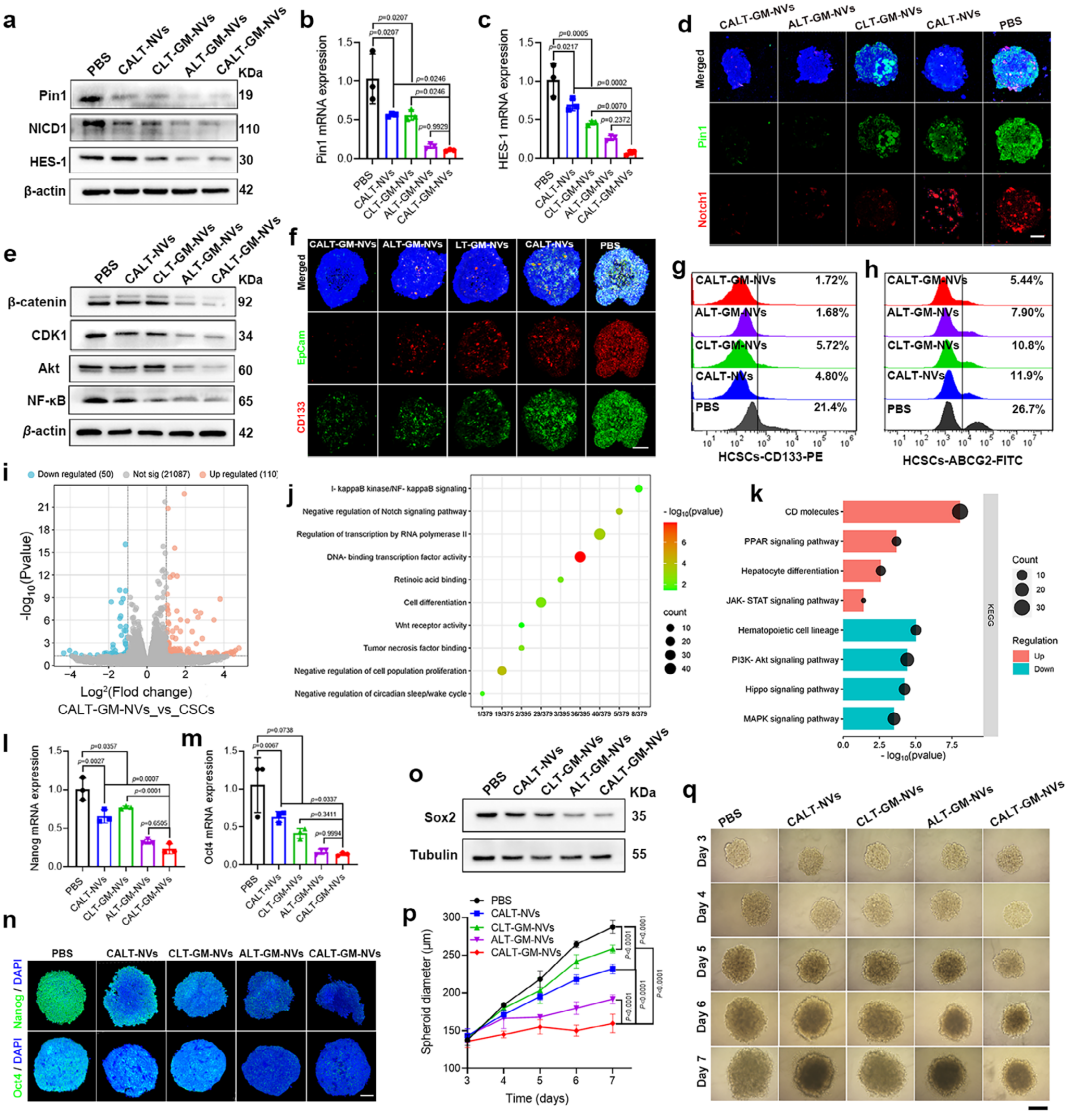
CALT‐GM‐NVs induced CSC differentiation by synergistically inhibiting Pin1 and Notch1. a) Immunoblotting detection of Pin1, NICD1, and HES‐1 protein levels in HCSCs after different treatments for 48 h. The relative mRNA level of Pin1 b) and HES‐1 c) in HCSCs (*n* = 3). d) Representative CLSM images of the Pin1 (green) and Notch1 (red) expression in HCSC tumorspheres (DAPI: blue). e) Immunoblotting detection of multiple oncoproteins in HCSCs following indicated treatments. f) Representative CLSM images of the CD133 (red) and EpCam (green) expression in HCSC tumorspheres (DAPI: blue). The percentage of CD133^+^ g) and ABCG2^+^ h) populations in HCSCs was determined by flow cytometry. i) Volcano plot of differentially expressed genes in HCSCs. j) GO pathway enrichment analysis in HCSCs. k) KEGG enrichment analysis in CSCs. l) Representative CLSM images of the Nanog and Oct4 expression in HCSC tumorspheres (DAPI: blue). The relative mRNA level of Nanog m) and Oct4 n) in HCSCs (*n* = 3). o) Immunoblotting detection of Sox2 levels in HCSCs. Representative images p) and quantitative analysis q) of HCSC tumor spheres formation from day 3 to day 7 under different treatments (*n* = 5). *p* Values were calculated by using one‐way ANOVA. Scale bars: 100 µm. All primer sequences are listed in Table S5 (Supporting Information). Scale bars: 100 µm.

Encouraged by these findings, we explored the effects of various nanovesicles on Pin1 and Notch1 signaling. As shown in **Figures** **4**a and S13 (Supporting Information), treatment with CALT‐NVs led to a noticeable reduction in both Pin1 levels and NICD1 release. CLT‐GM‐NVs effectively blocked Notch1 release, resulting in decreased expression of both HES‐1 and Pin1. Notably, ALT‐GM‐NVs and CALT‐GM‐NVs almost completely suppressed the activity of both Pin1 and Notch1 in HCSCs, causing a significant downregulation of HES‐1. Further confirming these findings, qRT‐PCR analysis showed that CALT‐GM‐NVs‐treated HCSCs exhibited a 2.66‐ and 1.71‐fold reduction in Pin1 mRNA expression, as well as a 2.57‐ and 2.38‐fold decrease in HES‐1 mRNA expression compared to CALT‐NVs‐ and CLT‐GM‐NVs‐treated cells, respectively (Figure 4b,c). Immunofluorescence co‐staining revealed simultaneous reductions in Pin1 (green fluorescence) and Notch1 (red fluorescence) signals in the HCSC tumorspheres treated with CALT‐GM‐NVs (Figure 4d). Notably, consistent results were also observed in MCSCs (Figure S14a–c, Supporting Information), supporting the broader applicability of this differentiation strategy across CSC subtypes. Next, we evaluated the influence of CALT‐GM‐NVs on several oncogenic proteins regulated by Pin1 and Notch1. As shown in Figure 4e, both CALT‐NVs and CLT‐GM‐NVs significantly reduced the expression of β‐catenin,^[^ ^6^ ^]^ cyclin‐dependent kinase 1 (CDK1),^[^ ^22^ ^]^ Akt,^[^ ^23^ ^]^ and NF‐κB^[^ ^24^ ^]^ in HCSCs. However, the co‐loading of ATRA and GSI in CALT‐GM‐NVs markedly enhanced the suppression of these oncoproteins. These results collectively confirmed that CALT‐GM‐NVs could effectively target and accumulate in CSCs, synergistically inhibiting Pin1 and Notch1, thereby blocking multiple cancer‐driving pathways.

Building on the promising ability of CALT‐GM‐NVs to synergistically inhibit Pin1 and Notch1, we next examined their effect on CSC differentiation by assessing stemness‐related markers. Immunofluorescence staining revealed that, compared to the intense CD133 (red) and EpCaM (green) signals in PBS‐treated HCSCs, both markers were significantly downregulated in CALT‐NVs‐ or CLT‐GM‐NVs‐treated cells (Figure 4f). Notably, treatment with ALT‐GM‐NVs and CALT‐GM‐NVs resulted in an even more pronounced reduction, with CD133 fluorescence almost undetectable in the CALT‐GM‐NVs group, demonstrating the potent differentiation‐inducing effects of these ATRA‐ and GSI‐loaded nanovesicles. Flow cytometry confirmed these observations, showing the greatest reduction in the percentage of CD133^+^ cells in HCSC spheroids treated with ALT‐GM‐NVs or CALT‐GM‐NVs (Figure 4g). Furthermore, both nanovesicles significantly decreased the expression of ABCG2, a multidrug transporter highly expressed in HCSCs. This reduction suggests that ATRA and GSI were retained in the cells, effectively promoting differentiation by limiting ABCG2‐mediated drug efflux (Figure 4h). The consistent findings in MCSCs further reinforce the robustness and translational potential of our dual‐targeted differentiation strategy in inducing CSC differentiation (Figure S15d,e, Supporting Information). Transcriptomic analysis of HCSCs treated with CALT‐GM‐NVs further supported these findings (Figure 4i–k). GO and KEGG pathway enrichment analyses revealed significant activation of pathways involved in cell differentiation and apoptosis, as well as negative regulation of the cell cycle. Conversely, key oncogenic signaling pathways, including PI3K‐Akt, MAPK, and Hippo, were notably suppressed (Figure 4j,k). In line with these findings, stemness‐related gene expression was markedly downregulated following treatment. Specifically, Quantitative RT‐PCR analysis showed that both ALT‐GM‐NVs and CALT‐GM‐NVs almost completely suppressed *Nanog* and *Oct4* mRNA expression in HCSCs (Figure 4l,m). Immunofluorescence staining further confirmed reduced protein levels of Nanog and Oct4 (Figure 4n), while western blot analysis revealed a significant decrease in Sox2 expression (Figure 4o). Collectively, these results highlight the enhanced ability of CALT‐GM‐NVs to disrupt CSC‐associated transcriptional programs, suppress stemness, and induce differentiation in HCSCs.

To further evaluate the functional effects of CSC differentiation, we monitored the growth of CSC tumorspheres. Spheroids treated with PBS rapidly expanded from ≈150 to ≈300 µm by day 7, reflecting the high tumorigenic potential of CD133^+^ HCSCs (Figure 4o). In contrast, tumorsphere growth was significantly inhibited in nanovesicle‐treated groups, with CALT‐NVs and CLT‐GM‐NVs‐treated HCSC spheroids reaching diameters of ≈231 and ≈258 µm, respectively, by day 7 (Figure 4p). CALT‐GM‐NVs exhibited the strongest inhibition, likely due to their combined Pin1 and Notch1 blockade. Although ALT‐GM‐NVs also contained ATRA and GSI, their inhibition of tumor sphere growth was less effective, likely due to the lack of specific CSC targeting, which reduced drug bioavailability. Together, these results demonstrated that targeted and synchronized delivery of ATRA and GSI to CSCs could effectively induce CSC differentiation in vitro.

### 2.5 Radiosensitization of CALT‐GM‐NVs to CSCs

**Figure 5:**
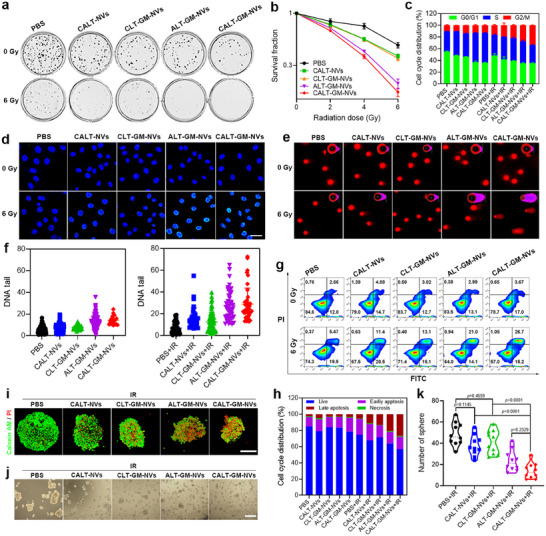
In vitro radiosensitization of CALT‐GM‐NVs to CSCs. a) Colony formation of HCSCs after the treatment with various formulations with or without 6 Gy of radiation. b) Clonogenic survival curves of HCSCs after the treatment with various formulations with 0, 2, 4, or 6 Gy of radiation (*n* = 6). c) Cell cycle distributions of HCSCs after different treatments (*n* = 3). d) Representative images of γ‐H2AX foci in HCSCs after indicated treatments (DAPI: blue). e) Representative images of DNA fragments in HCSCs after different treatments. f) Tail DNA ratio of HCSCs after different treatments under 0 or 6 Gy of radiation (*n* = 20). g,h) Flow cytometry analysis and quantification of the apoptosis of HCSCs after different treatments. i) Calcein AM/PI staining of HCSC spheroids after the treatment with various formulations under 6 Gy of radiation. Representative images j) and quantitative analysis k) of HCSC tumorspheres with indicated treatments on day 7 (*n* = 10). *p* Values were calculated by using one‐way ANOVA. Scale bars: 100 µm.

After confirming the potential of CALT‐GM‐NVs to promote CSC differentiation, we next evaluated their radiosensitization effects on HCC. To begin with, we assessed the cytotoxicity of various nanovesicles in the absence of irradiation on HCC cells, normal stem cells, and normal liver cells. The results showed that all formulations exhibited negligible toxicity toward human normal liver LO2 cells, mouse L929 fibroblasts, and normal stem cells (murine bone marrow‐derived mesenchymal stem cells, BM‐MSCs), demonstrating excellent biocompatibility (Figure S15, Supporting Information). CALT‐GM‐NVs slightly inhibited HepG2 cell proliferation, with cell viability dropping below 80% at an ATRA concentration of 10 µg mL^−1^ and a GSI concentration of 8.7 µg mL^−1^. Next, we explored the radiotherapy amplification effect of these nanovesicles on CSCs using the “gold standard” clonogenic assay. As shown in **Figures** **5**a and S16 (Supporting Information), HCSCs treated with CALT‐NVs or CLT‐GM‐NVs displayed similar trends in colony reduction, while ALT‐GM‐NVs had a more substantial inhibitory effect. Notably, CALT‐GM‐NVs combined with IR showed the greatest reduction in colony formation, indicating the strongest radiosensitization effect among the tested materials. The sensitizer enhancement ratio (SER) of CALT‐GM‐NVs was calculated to be 2.11, significantly higher than that of CALT‐NVs (1.14), CLT‐GM‐NVs (1.18), and ALT‐GM‐NVs (1.99) (Figure 5b). To confirm the broader applicability of this strategy, we tested CALT‐GM‐NVs‐mediated radiosensitization in MCSCs, which have enhanced tumorigenic and metastatic abilities. The CALT‐GM‐NVs + IR group showed a significant reduction in MCSC clonogenicity, with an SER value of 1.88 (Figure S17a,b, Supporting Information), supporting the effectiveness of this dual‐targeted approach across different CSC subtypes.

To understand the mechanism behind this radiosensitization, we analyzed the cell cycle distribution of HCSCs before and after IR using flow cytometry. CALT‐GM‐NVs treatment significantly decreased the radiation‐insensitive G0/G1 phase and increased the proportion of cells in the radiosensitive G2/M phase (Figure 5c; Figure S18, Supporting Information), contributing to the increased susceptibility of CSCs to radiotherapy. We further evaluated DNA damage using γ‐H2AX immunofluorescence and the comet assay. Without IR, almost no γ‐H2AX foci were detected. After radiation, distinct γ‐H2AX foci were observed in all nanovesicle‐treated groups, with the CALT‐GM‐NVs+IR group displaying the highest density of foci, indicating the most severe DNA double‐strand breaks (Figure 5d; Figure S17c, Supporting Information). The comet assay confirmed these findings, showing the longest tails in the CALT‐GM‐NVs+IR group, further supporting the radiosensitization effect of CALT‐GM‐NVs (Figure 5e,f). Moreover, CALT‐GM‐NVs pretreatment combined with radiotherapy led to the highest apoptosis rate of HCSCs among all the groups with radiation (Figure 5g,h). Consistently, calcein AM/PI staining also showed the strongest red fluorescence of apoptotic cells in HCSC spheres treated with CALT‐GM‐NVs (Figure 5i), indicating that CALT‐GM‐NVs can amplify radiation‐induced apoptosis. In addition, the tumorsphere disruption assay further highlighted the radiosensitization capacity of CALT‐GM‐NVs, which reduced the average colony number from 48.6 to 14.0 (Figure 5j,k). Collectively, these results demonstrate that CALT‐GM‐NVs effectively enhance the radiosensitivity of CSCs, providing a strong rationale for evaluating their radiosensitization potential in vivo.

### 2.6 Radiosensitization of CALT‐GM‐NVs to Cell Line‐Derived HCC Tumor Models

Before treatment, the possible side effects of each biomimetic nanovesicle were evaluated. No apparent hemolysis was observed in each group, and the hemolysis ratios were all below 5% even when high concentrations of nanovesicles (10 mg mL^−1^) were incubated with erythrocytes (Figure S19a,b, Supporting Information). The long‐term in vivo safety of these nanovesicles was evaluated in healthy mice with two administrations on days 0 and 7. On day 14, blood samples were collected from each mouse for hematological and blood biochemical analysis. As shown in Figure S19c (Supporting Information), all the key parameters of liver function, renal function, and hematology were within normal ranges and changed indistinctively before and after administration, confirming the excellent biological safety of the designed materials for in vivo applications. The accumulation of radiosensitizers in tumors was essential for exerting their functions. Therefore, we then evaluated the tumor targeting and accumulation of CALT‐GM‐NVs in HepG2 liver tumor‐bearing mice. After intravenous injection of DiR‐labeled CALT‐GM‐NVs or ALT‐GM‐NVs, CALT‐GM‐NVs rapidly accumulated in tumor tissues within 2 h and reached maximum retention at 12 h post‐injection (Figure S20a, Supporting Information). Quantitative analysis of fluorescence intensities in tumor regions, the peak accumulation of CALT‐GM‐NVs was 1.35 times higher than that of ALT‐GM‐NVs (Figure S20b, Supporting Information), emphasizing the crucial role of CP in the CSC targeting and tumor homing. At 12 h post‐injection, immunofluorescence staining of tumor sections revealed clear co‐localization of DiR signals with CD133⁺ cells (Figure S20c, Supporting Information), indicating selective accumulation of CALT‐GM‐NVs in CSC‐enriched regions.

**Figure 6:**
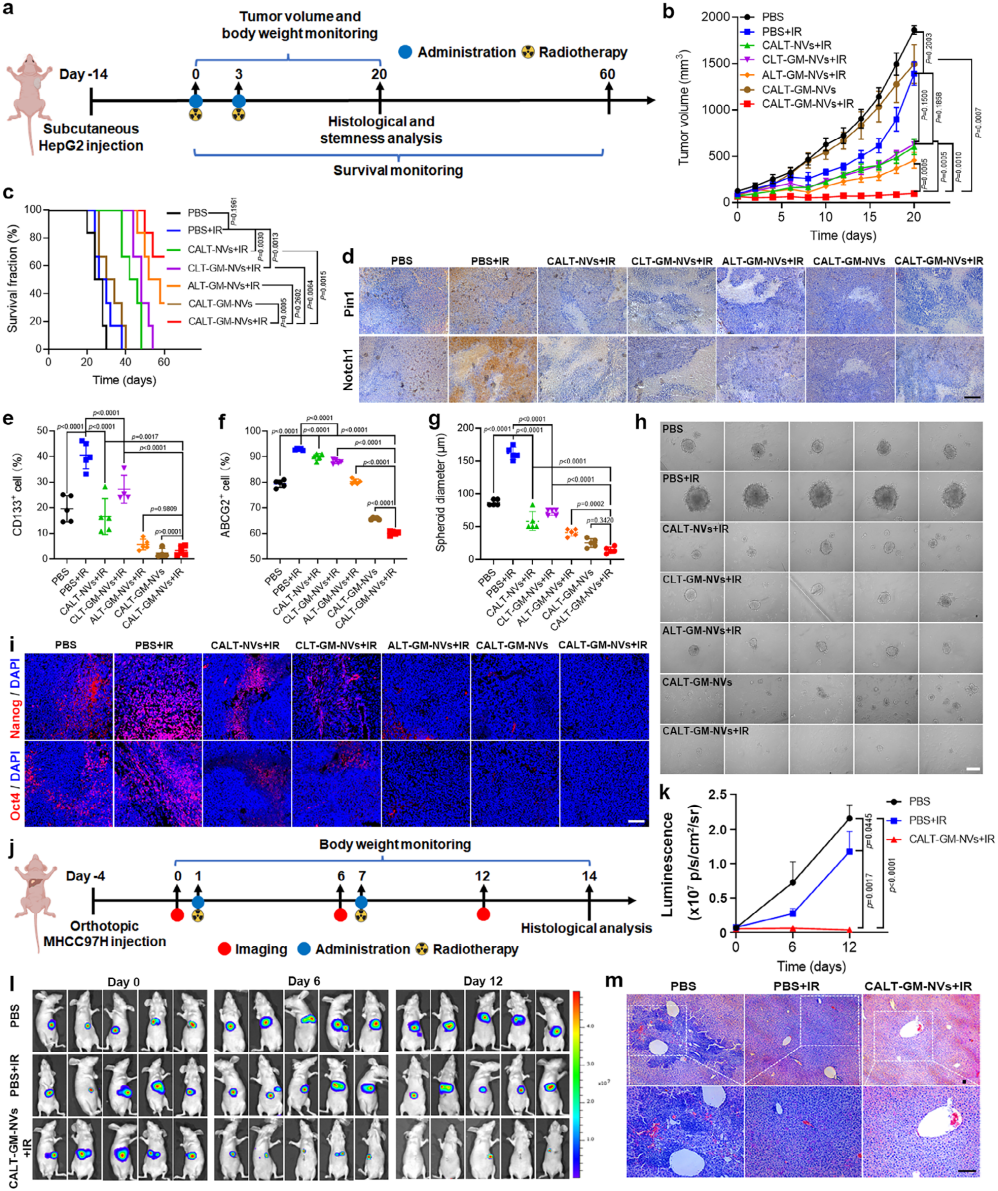
In vivo radiosensitization of CALT‐GM‐NVs to cell line‐derived HCC xenograft models. The treatment protocol for HepG2 subcutaneous a) and MHCC97H orthotopic j) tumor‐bearing mice. Tumor volumes (*n* = 5) b) and survivals (*n* = 6) c) of the HepG2 liver tumor‐bearing mice. Pin1 and Notch1 d) and Nanog and Oct4 i) expressions in the HepG2 tumor slices after indicated treatments. Flow cytometry analysis of the percentage of CD133^+^ e) and ABCG2^+^ f) cells in the HepG2 tumors after various treatments (*n* = 5). Representative images h) and quantitative analysis g) of tumorspheres formed by the isolated tumor cells from the HepG2 tumors after indicated treatments (*n* = 5). In vivo bioluminescence images l) and quantitative analysis k) of the MHCC97H orthotopic tumor‐bearing mice at different time points (*n* = 5). m) H&E staining of the MHCC97H tumor tissues on day 14. *P* values were calculated by using two‐tailed unpaired Student's *t*‐test (b), log‐rank tests (c), one‐way ANOVA (e,f,g), and two‐way ANOVA (k). Scale bars: 100 µm.

Next, we explored whether the CALT‐GM‐NVs could synergize with radiotherapy to effectively relieve tumor burdens. HepG2 liver tumor‐bearing mice with a tumor volume of ≈100 mm^3^ were randomly assigned into 7 groups and injected twice with different formulations via the tail vein on day 0 and 3, respectively, followed by 6 Gy of radiation or not at 12 h post‐administration (**Figure** **6**a). Compared with the rapidly proliferating tumors in the PBS group, the tumors with PBS+IR treatment showed limited delay of tumor growth, which may be due to the extensive infiltration of the inherent and radiation‐induced radioresistant HCSCs (Figure 6b; Figure S21a, Supporting Information). CALT‐NVs+IR and CLT‐GM‐NVs+IR showed moderate tumor growth inhibition, suggesting that both ATRA and GSI alone could assist radiotherapy. Notably, CALT‐GM‐NVs pretreatment with radiotherapy exhibited the most significant tumor growth suppression, leading to a much smaller final tumor volume than ALT‐GM‐NVs+IR treatment, which was probably attributed to the preferable pharmacokinetic properties of CALT‐GM‐NVs. To validate the therapeutic efficacy, tumor tissues from each group were collected for histological analysis. Hematoxylin and eosin (H&E) staining revealed typical apoptotic features, such as nuclear condensation and cell morphological changes, in the tumors receiving radiotherapy. Among all the groups, CALT‐GM‐NVs+IR caused the most extensive and severe apoptosis of tumor cells, confirming the excellent antitumor performance of this combination therapy (Figure S21b, Supporting Information). This antitumor effect was further supported by the TdT­mediated dUTP Nick‐End Labeling (TUNEL) staining (Figure S21c, Supporting Information), indicating that CALT‐GM‐NVs were a competent radiosensitizer capable of boosting radiotherapy‐induced apoptosis. The commendable tumor suppression of CALT‐GM‐NVs with radiotherapy significantly prolonged the lifetime of tumor‐bearing mice, with a 60‐day survival rate of 66.7% (Figure 6c). Moreover, all the mice showed no appreciable changes in body weight during treatment (Figure S21d, Supporting Information), and H&E staining of the main organs also showed no discernible pathological lesions (Figure S22, Supporting Information), demonstrating the favorable biosafety of these treatment modalities.

Given the efficient inhibition of CALT‐GM‐NVs on tumor growth, we wondered whether this nanovesicle attenuated the stemness‐related phenotype of tumor cells in vivo. Immunohistochemical analysis in Figure 6d showed that the expression of Pin1 and Notch1 was remarkably upregulated after radiotherapy. However, the introduction of ATRA‐ and/or GSI‐loaded nanovesicles before radiation greatly inhibited the elevation of both Pin1 and Notch1 levels. CALT‐GM‐NVs induced the most significant downregulation of Pin1 and Notch1, suggesting the optimal in vivo CSC differentiation effect of this biomimetic nanovesicle. Subsequently, we investigated the populations of intratumoral CSCs after various treatments. Flow cytometry analysis showed that the proportion of CSC markers CD133^+^, ABCG2^+^, and CD24^+^ was dramatically augmented by radiotherapy, while CALT‐GM‐NVs pretreatment dramatically abolished this expansion of CSCs (Figure 6e,f; Figure S23, Supporting Information). Moreover, the isolated tumor cells from these mice after radiotherapy alone exhibited enhanced tumor‐sphere formation performance, with an average spheroid diameter of 158.3 µm after 7 days of culture, which was much larger than those in the PBS group (Figure 6g,h). Fortunately, this radiation‐promoted CSCs expansion can be profoundly abrogated by our biomimetic nanovesicles, and CALT‐NVs, CLT‐GM‐NVs, and ALT‐GM‐NVs treatment led to a 2.77‐, 2.25‐, and 3.95‐fold retardation in the tumorsphere growth, respectively. Notably, inconspicuous tumor spheroids were observed in the CALT‐GM‐NVs and CALT‐GM‐NVs+IR groups, suggesting that CALT‐GM‐NVs can serve as a promising candidate for eliminating CSCs to overcome radioresistance. Immunohistochemical staining for CSC markers, such as Nanog and Oct4, also supported these findings (Figure 6i).

After confirming the radiosensitization of CALT‐GM‐NVs, we transplanted luciferase‐expressed MHCC97H cells into livers to build orthotopic HCC models for further investigating the antitumor efficiency of this nanovesicle with radiotherapy. After 4 days of inoculation with MHCC97H cells, the tumors were monitored through bioluminescence imaging to confirm the successful establishment of tumor models (Figure 6j). Then, tumor‐bearing mice were randomly divided into 3 groups and injected twice with PBS or CALT‐GM‐NVs via the tail vein on days 1 and 7, followed by 6 Gy of radiation or not at 12 h post‐administration. The bioluminescence signals in the PBS and PBS+IR groups intensified over time, and tumor foci were evident in the livers of all mice on day 14 (Figure 6k,l). However, the bioluminescence signals in the tumor areas of the CALT‐GM‐NVs+IR‐treated mice gradually diminished with time and were almost undetectable 2 weeks after the treatment. Histological examination confirmed the existence of tumor foci in the livers, and CALT‐GM‐NVs combined with radiotherapy significantly inhibited tumor progression (Figure 6m). The body weight of mice receiving various treatments showed little change, further validating the safety of this combination therapy (Figure S24, Supporting Information). These data substantiated that CALT‐GM‐NVs contributed to improving the radiotherapy efficacy of cell line‐derived HCC tumors by inducing the differentiation of CSCs.

### 2.7 Radiosensitization of CALT‐GM‐NVs to Patient‐Derived HCC Tumor Models

**Figure 7:**
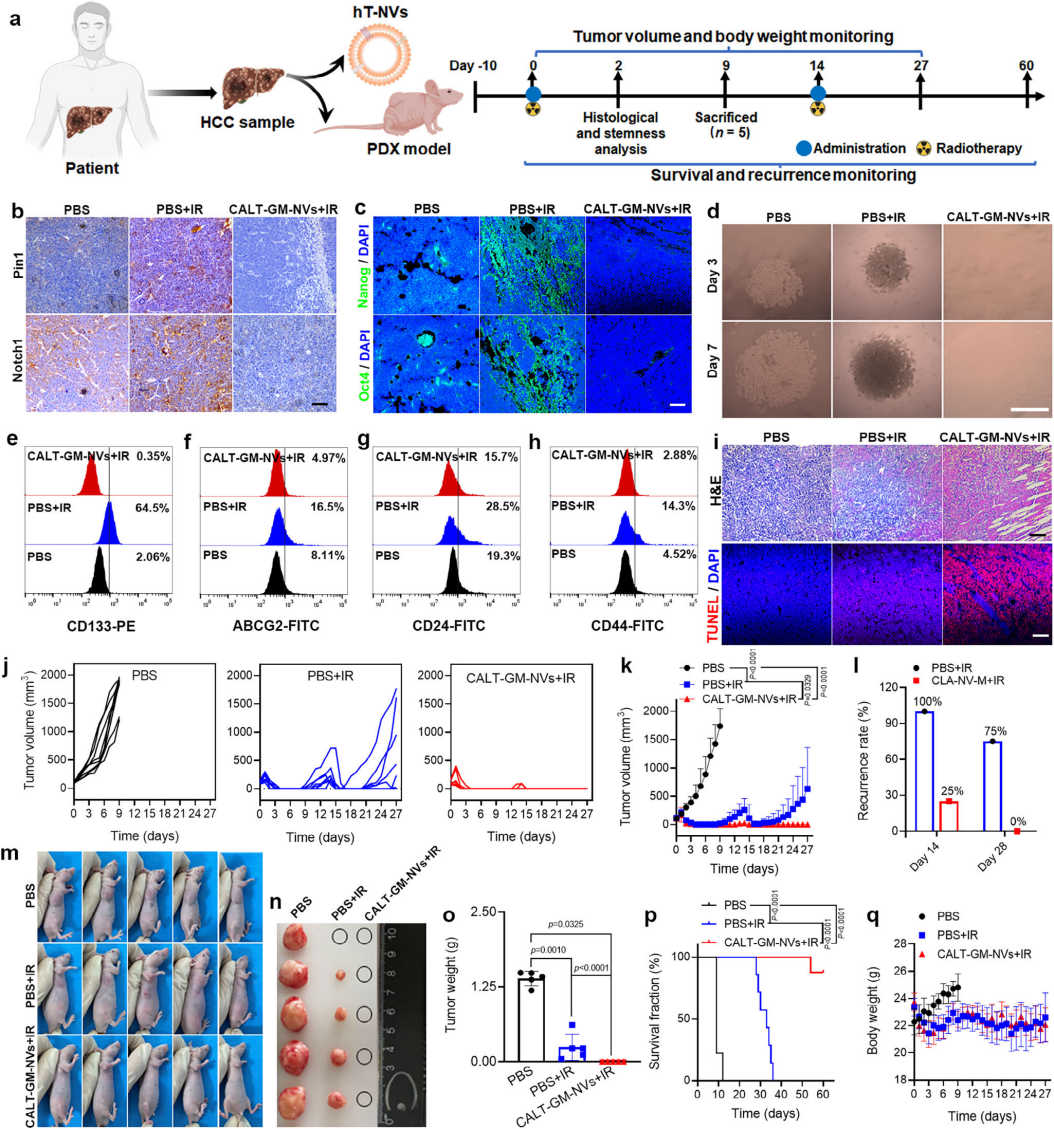
Radiosensitization of CALT‐GM‐NVs to patient‐derived HCC xenograft models. a) The treatment protocol for PDX tumor‐bearing mice. Pin1 and Notch1 b) and Nanog and Oct4 c) expressions in the PDX tumor slices after indicated treatments. d) Representative images of tumor spheres formed by the isolated tumor cells from PDX tumors after indicated treatments (*n* = 5). Flow cytometry analysis of the percentage of CD133^+^ e), ABCG2^+^ f), CD24 g), and CD44 h) cells in the PDX tumors after various treatments (*n* = 5). i) H&E and TUNEL staining of the PDX tumor tissues on day 2. Individual j) and overall k) tumor growth curves of the PDX tumor‐bearing mice with different treatments (*n* = 8). l) The recurrence rate of the PDX tumor‐bearing mice after the treatment with PBS+IR or CALT‐GM‐NVs+IR (*n* = 8). Representative photographs of the PDX tumor‐bearing mice m) as well as the photographs n) and weights o) of the excised PDX tumors on day 9 (*n* = 5). Survival p) and body weights q) of the PDX tumor‐bearing mice with different treatments (*n* = 8). *p* Values were calculated by log‐rank tests (k,o) and two‐tailed unpaired Student's *t*‐test (p). Scale bars: 100 µm.

Patient‐derived xenografts (PDX) faithfully retain the histological and molecular characteristics of the original tumors, making them a robust preclinical model for evaluating therapeutic efficacy in a clinically relevant context.^[^ ^25^ ^]^ To assess the translational potential of CALT‐GM‐NVs, we constructed a humanized delivery system using nanovesicles derived from human HCC tumor cell membranes (hT‐NVs) and evaluated its radiosensitizing effects in HCC PDX models. The hT‐NVs were isolated from HCC tumor specimens using the same protocol as previously described, and successful fusion with CALT‐GM‐NVs was confirmed by TEM (Figure S25, Supporting Information). Another portion of HCC tumor specimens was subcutaneously transplanted into the highly immunodeficient mice after three successive passages, and when the tumor size reached approximately 100 mm^3^, the mice received different treatments (**Figure** **7**a). To validate the therapeutic relevance of our targets within the established PDX model, we first assessed the baseline expression levels of Pin1 and Notch1 in PDX tumor tissues compared to normal liver. Immunohistochemical staining revealed markedly elevated expression of both proteins in the PDX tumors relative to normal liver tissue (Figure S26, Supporting Information), confirming that the patient‐derived tumors preserved the oncogenic characteristics of Pin1 and Notch1 overexpression. Following a single treatment cycle, tumors in the PBS+IR group showed marked upregulation of Pin1 and Notch1 expression, whereas combined treatment with CALT‐GM‐NVs and irradiation substantially suppressed their levels (Figure 7b). Consistently, immunohistochemical analysis of Nanog and Oct4 showed similar expression patterns, with CALT‐GM‐NVs inducing a pronounced reduction in these stemness markers, thereby confirming their potent capacity to eliminate cancer stem‐like cells in vivo (Figure 7c).

Then, tumor cells were isolated from the PDX tumors after different treatments for tumor sphere formation evaluations and the intratumoral CSC fractions. As shown in Figure 7d and Figure S27a (Supporting Information), the tumor cells from PBS+IR‐treated mice formed solid spheroids with larger diameters than tumorspheres in the PBS group. However, CALT‐GM‐NVs pretreatment significantly inhibited the formation of tumor spheres because the inherent and IR‐induced CSCs were differentiated by this nanovesicle. Moreover, the percentage of CD133^+^, ABCG2^+^, CD24^+^, and CD44^+^ cells in the tumors treated with CALT‐GM‐NVs+IR was 0.35%, 4.97%, 15.7%, and 2.88%, respectively, which were much lower than those (64.5%, 16.5%, 28.5%, and 14.3%, respectively) of the PBS+IR group (Figure 7e–h; Figure S27b, Supporting Information). H&E and TUNEL staining revealed that CALT‐GM‐NVs cooperated with IR, resulting in the most significant apoptosis in tumor tissues, foreboding that this combination therapy would achieve decent treatment outcomes for PDX models (Figure 7i). Only 9 days after starting treatment, most tumors in the PBS group were close to 2000 cm^3^, and thus, we stopped recording the tumor volume of these PBS‐treated mice (Figure 7j,k). For the mice in the PBS+IR group, their tumors all visually disappeared on day 4 post‐radiation, while gradually recurred from day 9, and the recurrence rate was 100% on day 14 (Figure 7l; Figure S27c, Supporting Information). These results indicated the extremely high degree of malignancy of this patient‐derived tumor. The tumors of the CALT‐GM‐NVs+IR‐treated mice also gradually shrank and eventually vanished within 4 days, and only two tumors experienced recurrence on days 13 and 14, respectively (Figure 7m–o). On day 9, 5 mice in each group were sacrificed, and their tumors were isolated for photographing and weighing. The tumors in the PBS group were quite big, and most of the tumors in the PBS+IR group indeed experienced recurrence. However, no tumor was found in the CALT‐GM‐NVs+IR group on this day. For the relapsed mice in the PBS+IR (*n* = 8) and CALT‐GM‐NVs+IR (*n* = 2) group, they received the second treatment on day 14, and the tumor volume and mouse weight of these mice and the relapse‐free mice in the CALT‐GM‐NVs group (*n* = 6) were continued to be recorded. As can be seen, most of the tumors in the PBS+IR group expanded rapidly after a brief decline, with 75% of the tumors returning on day 28, while no tumor was observed in the mice of the CALT‐GM‐NVs+IR group on this day. Notably, all mice in the PBS and PBS+IR groups died within 12 and 36 days after the beginning of the treatment, respectively, while seven of eight mice in the CALT‐GM‐NVs+IR group still survived until day 60 (Figure 7p). In addition, the body weight of the mice receiving radiotherapy, regardless of CALT‐GM‐NVs administration, showed similar changes, as slight weight loss in the few days following radiation and gradual weight recovery, once again validating the favorable biosafety of this biomimetic nanovesicle (Figure 7q). Together, these results further demonstrated that CALT‐GM‐NVs could smoothly induce the differentiation of CSCs in the PDX tumors, and thus effectively enhance the radiosensitivity of HCC.

## 3 Discussion

The widespread radioresistance of tumor cells has seriously impaired the efficacy of radiotherapy. Despite the development and utilization of diverse radiosensitizers in recent years, the dilemma of the low sensitivity of tumors to radiotherapy has not yet been broken through till now.^[^ ^26^ ^]^ This issue is primarily attributed to tumor heterogeneity, which results in considerable differences in the radiation response among various tumor subpopulations, thereby endowing tumor cells with tolerance to radiotherapy. CSCs are recognized as the “seeds” of driving tumor heterogeneity and are therefore considered to be the source of tumor resistance to radiotherapy. Furthermore, quiescent CSCs can be “awakened” by radiotherapy, leading to an enrichment of CSC populations.^[^ ^27^ ^]^ Concurrently, normal tumor cells with high malignant potential may undergo “awakening” through radiation‐induced dedifferentiation, acquiring the CSC phenotype.^[^ ^28^ ^]^ Our study also provides supporting evidence that the expression of stemness factors *Nanog*, *Oct4*, and *Sox2* is upregulated in RT‐treated CSCs and tumor models, further aggravating radioresistance. Consequently, eliminating CSCs holds great potential to achieve radiosensitization.

Recent research suggests that the Notch1 signaling pathway plays an important role in maintaining CSC phenotypes.^[^ ^12^, ^29^ ^]^ Furthermore, the crosstalk with other signaling pathways may influence the overall effect of the Notch1 signaling pathway. Among these, Pin1 has been reported as a potential co‐regulator that modulates Notch1 activity via post‐translational mechanisms in other cancer contexts.^[^ ^19^ ^]^ In this study, we explored the relationship between Pin1 and Notch1 in HCC using both TCGA transcriptomic data and clinically derived HCC samples and observed their concurrent upregulation. Although co‐expression alone cannot confirm direct interaction, it supports their potential functional synergy. Moreover, we conducted a series of in vitro and in vivo experiments that demonstrate mutual reinforcement between Pin1 and Notch1 expression and confirm that activation of this axis contributes to CSC maintenance and radioresistance in HCC. While previous studies have primarily focused on targeting Pin1 or Notch1 individually, these approaches may be limited due to compensatory activation of parallel pathways and the inherent plasticity of CSCs.^[^ ^11^, ^30^ ^]^ To our knowledge, this is the first study in HCC to functionally validate a dual‐targeting strategy that simultaneously inhibits both Pin1 and Notch1. Our data demonstrate that this combinatorial approach is significantly more effective in reducing CSC stemness and enhancing radiosensitivity compared to either single‐agent treatment alone. These findings highlight the critical limitations of current monotherapies and provide strong preclinical rationale for dual‐target inhibition as a more robust and durable strategy, with potential applicability to other CSC‐enriched solid tumors.

Biomimetic drug delivery nanosystems based on tumor cell membranes have attracted extensive attention in recent years due to their simple preparation, decent biocompatibility, low immunogenicity, and high blood circulation stability. Furthermore, the presence of membrane surface substances, such as N‐cadherin, galactin‐3, and epithelial cell adhesion molecules, endows membrane‐based nanomedicines with the ability to recognize homotypic tumors.^[^ ^31^ ^]^ Additionally, most membrane materials can be stored at low temperatures for a long time, providing convenience for their application and reprocessing. These combined benefits inspire us to utilize membrane materials in the construction of biomimetic nanocarriers for the synchronous delivery of ATRA and GSI to fulfill the synergistic inhibition of Pin1 and Notch1. Our results also demonstrate the excellent CSC differentiation performance and clinical radiosensitization potential of the fabricated biomimetic drug delivery nanosystems.

This study revealed the synergistic role of Pin1 and Notch1 in maintaining the stemness of CSCs and radiotherapy tolerance and developed a nano‐formulation targeting CSCs to induce differentiation. While this nano‐formulation primarily targets CD133^+^ CSCs, which are commonly found in HCC, the inherent heterogeneity of CSCs may potentially affect targeting efficiency. Additionally, the exact mechanism behind the synergism of Pin1 and Notch1 remains to be fully elucidated, and further research will be necessary to clarify these interactions. Although the nano‐formulation demonstrated promising therapeutic efficacy in PDX models, the sample size was limited and may not fully represent all HCC types. In the future, optimizing the nano‐formulation based on patients' genetic profiles and CSC heterogeneity will be a key challenge for clinical translation.

## 4 Experimental Section

### Study Approval

All animal experiments were conducted following the protocols approved by the Animal Ethical and Welfare Committee (AEWC) of the Institute of Radiation Medicine, Chinese Academy of Medical Sciences & Peking Union Medical College (IRM‐DWLL‐2023065). A primary tumor sample was acquired from an HCC volunteer (age 32, female), who was recruited through the protocol at the National Cancer Center/National Clinical Research Center for Cancer/Cancer Hospital, and patient sample‐associated experiments were approved by the Ethics Committee, Cancer Hospital, Chinese Academy of Medical Sciences (201100661‐CRC).

### Clinical Samples and Analysis

A tissue microarray (TMA, HLivH165Su01) consisting of 86 liver cancer pairs and 79 corresponding adjacent nontumor liver tissues was purchased from Outdo Biotech Co., Ltd. (Shanghai, China). Immunohistochemistry analysis of Pin1 (Abcam) and activated Notch1 (Abcam) was performed according to the manufacturer's instructions, and the sections were imaged through an automatic multispectral imaging system (Vectra II, PerkinElmer version 2.0.7.1). The results were analyzed by Image J. Positive staining was assessed using a semiquantitative scoring system: 0 (no staining), 1 (low staining), 2 (moderate staining), and 4 (strong staining). 0–1 was defined as negative, while 2–4 was defined as positive.

Primary HCC cells were isolated from patient tumor tissues by enzymatic digestion and cultured under sphere‐forming conditions. To mimic clinical radiotherapy, cells were first exposed to 6 Gy irradiation. After 48 h, a second irradiation was administered at varying doses (0, 2, 4, 6 Gy), followed by clonogenic assays to evaluate radioresistance.

### Construction of HCC Cells with Pin1, Notch1 or Fbxw7α Overexpression or Knockdown

Overexpression experiments: Plasmids harboring the human Pin1, NICD1, or Fbxw7α coding sequences were constructed by GENEWIZ (Suzhou, China) using the pcDNA3.1 vector. HepG2, Bel7402, and MHCC97H cells were transfected with pcDNA3.1‐Pin1, pcDNA3.1‐Notch1, or pcDNA3.1‐Fbxw7α using Lipofectamine 3000 reagent (Invitrogen) for 48 h. Transfected cells were selected in medium containing 200 µg mL^−1^ G‐418 (Sigma) for 10 days. The overexpression of Pin1 or Notch1 was confirmed by western bolt using anti‐human Pin1 antibody (1:1000; Abcam, ab192036), anti‐human cleaved Notch1 antibody (Val1744, D3B8; 1:1000; CST) or anti‐human Fbxw7α antibody (1:1000; ab192328) overnight at 4 °C.

Knockdown experiments: small interfering RNAs (siRNAs) targeting Pin1 (si‐P) and Notch1 (si‐N) were synthesized by OriGene Biotechnology (Shanghai, China). HepG2, Bel7402, and MHCC97H cells were transfected with the indicated siRNAs using Lipofectamine 3000 reagent according to the manufacturer's instructions. A non‐targeting siRNA was used as a negative control. Cells were harvested 48–72 h post‐transfection for further analyses. Knockdown efficiency was validated by western blot using the same antibodies as described above.

### RNA‐Seq

HepG2 cells and HCSCs were exposed to 6 Gy γ‐rays and continued to be cultured for 48 h to extract total RNA. HCSCs were co‐cultured with CALT‐GM‐NVs for 72 h, and total RNA was extracted. The high‐throughput sequencing was conducted in Novogene Bioinformatics Technology Co., Ltd. All differential gene expression analysis was conducted using DESeq2. R statistics packages were used for visualization and enrichment of the differential genes.

### CSCs Radiotherapy Resistance Evaluation

To analyze the effects of Pin1 and Notch1 on CSC radioresistance, 100 000 cells per well of CSCs were inoculated into 6‐well ultra‐low attachment plates and cultured in CSC medium for 48 h. The cells were then radiated with 6 Gy of γ‐rays and incubated for another 24 h. The expression of Pin1 or Notch1 was detected by Western blot as mentioned above. The gene expression of Pin1 and HES‐1 was analyzed by qRT‐PCR as described above.

To analyze the impact of Pin1, Notch1, or Fbxw7α on CSC tumorsphere resistance to radiation, CSCs subjected to different treatments (overexpression or knockdown of Pin1, Notch1, and co‐overexpression of Pin1 and Fbxw7α) were seeded at 1000 cells per well into 96‐well ultra‐low attachment plates and cultured for 7 days. Tumorspheres were then treated with ATRA, GSI, or their combination, or left untreated, for 48 h, followed by exposure to 6 Gy of γ‐rays and continued culture for another 24 h. Tumorspheres with diameters > ≈50 µm were counted under an optical microscope.

### CSCs Recognize and Binding Ability of CALT‐GM‐NVs

HCSCs were seeded at 1 × 10^5^ cells mL^−1^ into confocal dishes or 1000 cells per well were inoculated in 96‐well Nunclon Spheres plates. DiI‐labeled CALT‐GM‐NVs were dissolved in the medium at a concentration of 100 µg mL^−1^ (equivalent ATRA and GSI concentrations of 5 and 4.35 µg mL^−1^, respectively; the same concentrations were used across all in vitro experiments). After washing the cells with cold PBS, DiI‐labeled CALT‐GM‐NVs were added, along with FITC‐labeled anti‐CD133 (0.1 µm) and Hoechst 33 342 (10 mM). Binding was achieved by incubating the cells in the solution at 4 °C for 40 min. Cells were washed three times with cold PBS and then observed for binding under CLSM.

To investigate the recognition ability of CALT‐GM‐NVs on HCSCs and HepG2 cells were incubated with DiO‐labeled CALT‐GM‐NVs for 4 h, respectively. After washing 2 times with PBS, the cells were fixed with 4% paraformaldehyde (PFA) for 15 min. Then, the cytoskeleton was labeled with Actin‐Tracker Red‐Rhodamine (excitation/emission = 551/567 nm) for 40 min at room temperature after washing with PBS. Finally, the nuclei were negatively stained with DAPI. The samples were analyzed under CLSM.

### Evaluation of the Ability of CALT‐GM‐NVs to Induce CSCs Differentiation In Vitro—*Flow Cytometry Analysis of the CSC Subpopulation Proportions*

To analyze the differentiation of CSCs induced by different biomimetic nanovesicles, the population of CD133^+^ and ABCG2^+^ cells was analyzed by flow cytometry. Briefly, 30 000 cells per well of CSCs were inoculated into 6‐well ultra‐low attachment plates and cultured in CSC medium containing different biomimetic nanovesicles for 72 h. Subsequently, the single cell suspensions were collected and washed 3 times with PBS and incubated with PE anti‐human CD133 or FITC anti‐human ABCG2 for 30 min at 4 °C, respectively, and detected by flow cytometry.

### Evaluation of the Ability of CALT‐GM‐NVs to Induce CSCs Differentiation In Vitro—*Detection of Stemness Factor Expression*

The expression of stemness‐related factors (Nanog, Oct4, and Sox2) in CSCs after treatment with different biomimetic nanovesicles was analyzed by qRT‐PCR, Western blot, and immunofluorescence staining. For qRT‐PCR detection, 100 000 cells/well of CSCs were inoculated into 6‐well ultra‐low attachment plates and cultured in CSC medium containing different biomimetic nanovesicles for 72 h. Then, total RNA samples were extracted according to the manufacturer's protocols. The genes were expressed as 2‐(^△△^CT) after normalization by GAPDH. All primer sequences are listed in Table S5 (Supporting Information). For protein expression analysis, cells treated under the same conditions were lysed and subjected to Western blot. Primary antibodies against Sox2 (1:1000; CST; D9B8N) were used as described above. Tubulin was used as a loading control. For immunofluorescence staining, 1000 cells per well of CSCs were inoculated in 96‐well Nunclon Spheres plates for 72 h and treated under the same conditions. Then, the CSC tumorspheres were incubated with anti‐human antibodies Nanog (1:200; CST; 4903T) or Oct4 (1:1600; CST; C52G3) as described previously and observed under CLSM.

### Evaluation of the Ability of CALT‐GM‐NVs to Induce CSCs Differentiation In Vitro—*HCC Stem Cell Surface Marker Immunofluorescence Staining*

The expression of CD133 and EpCam in CSC spheres was observed by immunofluorescence staining. 1000 cells per well were inoculated in 96‐well Nunclon Spheres plates for 72 h and continued co‐incubated with biomimetic nanovesicles for another 48 h. Then, CSC tumorspheres were co‐incubated with anti‐human antibodies CD133 (1:200; Abcam) and EpCam (1:200; CST) overnight at 4 °C. After PBS washing, the spheres were incubated with anti‐rabbit AF488‐IgG antibody (1:200; Abcam) and anti‐mouse AF488‐IgG or AF594‐IgG (1:200; Abcam) for 1 h at room temperature. Then, the tumorspheres were negatively stained with DAPI and observed under CLSM.

### Evaluation of the Ability of CALT‐GM‐NVs to Induce CSCs Differentiation In Vitro—*Inhibition CSCs Self‐Renewal*

The tumor sphere formation assay was used to analyze the self‐renewal ability of CSCs after treatment with different biomimetic nanovesicles. Specifically, 1000 cells per well were inoculated into 96‐well ultra‐low attachment plates and cultured with serum‐free DMEM/F12 medium containing different biomimetic nanovesicles for 72 h. Then, 1000 cells per well of each group were re‐inoculated in 96‐well Nunclon Sphera plates and cultured for another 7 days. The diameters of the formed tumor spheres were observed and recorded under an optical microscope to analyze the effect of different mixed nanoparticle treatments on the ability of tumor sphere formation.

### Evaluation of the Ability of CALT‐GM‐NVs to Enhance CSC Radiosensitivity In Vitro—*Colony Formation Assay*

Briefly, the cells were incubated in 6‐well plates at a density of 500 cells per well for 12 h. They were then incubated with different biomimetic nanovesicles for another 48 h. The cells were then radiated with different doses (0, 2, 4, and 6 Gy) of γ‐rays and incubated for another 7 days. The forming colonies were stained with 0.25% crystalline violet. Survival rate and SER were calculated according to standard methods.

### Evaluation of the Ability of CALT‐GM‐NVs to Enhance CSC Radiosensitivity In Vitro—*DNA Breakage Detection*

DNA breaks were detected by the γ‐H2AX fluorescence signal and single‐cell gel electrophoresis. First, CSCs enriched in low‐adhesion 6‐well plates were incubated with different biomimetic nanovesicles for 72 h and then irradiated with 6 Gy of γ‐rays. After incubation at 37 °C for 1 h, each group of cells was collected, and the fluorescence signal of γ‐H2AX was detected according to the above immunofluorescence staining protocol. Second, single‐cell gel electrophoresis was used to detect DNA breaks. After agarose covering the slides 30 µL of cells and 70 µL of low melting point agarose were added, and the slides were lysed in lysis buffer for 2.5 h. Subsequently, the slides were electrophoresed for 20 min at 30 V in an electrophoresis solution. Neutralized for 20 min, followed by PBS washing, and then the slides were stained with ethidium bromide for comets. DNA damage was analyzed using the Comet Analysis Software Program (CASP).

### Evaluation of the Ability of CALT‐GM‐NVs to Enhance CSC Radiosensitivity In Vitro—*Cell Cycle Assay*

Cell cycle changes after treatment with biomimetic nanovesicles were detected by flow cytometry. CSCs were enriched in low‐adhesion 6‐well plates and incubated with different biomimetic nanovesicles for 72 h and then irradiated with 6 Gy of γ‐rays. After continuing incubation for 24 h, cells were collected and fixed with 70% ethanol overnight at 4 °C. Then, the cells were washed and incubated with RNase A for 30 min at 37 °C to remove the RNA. Finally, the samples were stained with 50 µg mL^−1^ of propidium iodide (PI, Ex/Em = 488 nm/630 nm), incubated for 10 min at 37 °C, and analyzed by flow cytometry.

### Evaluation of the Ability of CALT‐GM‐NVs to Enhance CSC Radiosensitivity In Vitro—*Cell Apoptosis Analysis*

The apoptosis levels of CSCs radiated with 6 Gy of γ‐rays after induction of differentiation by different biomimetic nanovesicles were detected using the Annexin V‐FITC/PI apoptosis assay kit. CSCs enriched in low‐adhesion 6‐well plates were incubated with different biomimetic nanovesicles for 72 h and then irradiated with 6 Gy of γ‐rays. After another 24 h of incubation, the cells were treated based on the instructions of the apoptosis detection kit and subsequently analyzed by flow cytometry.

For tumor spheroids, Calcein‐AM/PI staining, 1000 CSC per well enriched in low‐adhesion 96‐well plates, were incubated with different biomimetic nanovesicles for 72 h and then irradiated with 6 Gy of γ‐rays. After another 24 h of incubation, the tumor spheroids were simultaneously stained with Calcein‐AM (1 mM) and PI (0.1 mM) for 10 min and observed by CLSM.

### Evaluation of the Ability of CALT‐GM‐NVs to Enhance CSC Radiosensitivity In Vitro—*Tumorspheres Destruction Assay*

A total of 1000 cells per well were inoculated into 96‐well ultra‐low attachment plates, and HCSCs tumor spheres with diameters >≈50 µm were incubated with different biomimetic nanovesicles for 72 h. After 6 Gy of radiation, the cells were cultured for another 24 h, and then the number of tumor spheres with diameters >≈50 µm was recorded under a light microscope.

### In Vivo Antitumor Efficacy of CALT‐GM‐NVs with Radiotherapy Against Subcutaneous HCC Models—*Tumor Targeting*

After establishing HepG2 subcutaneous tumor models, the mice with a tumor volume ≈100 mm^3^ were intravenously injected with 100 µL of DiR‐labeled CALT‐GM‐NVs or ALT‐GM‐NVs. The in vivo distribution of these two nanovesicles was imaged by an In Vivo Master Imaging System at 2, 4, 8, 12, 24, 48, and 72 h post‐injection. The fluorescence images were analyzed using Living Image 3.1 (Caliper Life Sciences). To evaluate tumor‐targeting specificity, tumor tissues were harvested 12 h after injection, embedded with frozen O.C.T., and cut into 8 µm slices. Immunofluorescence staining was performed using anti‐CD133 antibody and AF488‐IgG secondary antibody, followed by confocal imaging.

### In Vivo Antitumor Efficacy of CALT‐GM‐NVs with Radiotherapy Against Subcutaneous HCC Models—*Therapeutic Effect*

The HepG2 subcutaneous tumor‐bearing mice are randomly divided with a tumor volume ≈100 mm^3^ into 7 groups (11 mice per group), including the PBS, PBS+IR, CALT‐NVs+IR, CLT‐GM‐NVs+IR, ALT‐GM‐NVs+IR, CALT‐GM‐NVs, and CALT‐GM‐NVS+IR groups. Mice were injected via the tail vein with 100 µL of PBS or various nanovesicle solutions (equivalent ATRA and GSI concentrations of 10 and 8.7 µg mL^−1^, respectively). At 12 h post‐administration, the mice, except for those in the PBS group, were irradiated with 6 Gy of γ‐rays. All the mice were treated twice on days 0 and 3. Tumor volumes and body weights of 5 mice in each group were monitored every other day over 21 days. On day 20, the 5 mice in each group were sacrificed to collect tumors and major organs (heart, liver, spleen, lung, and kidney) for histological analysis. Meanwhile, the tumor cells were isolated from these tumors for CSC marker detection by flow cytometry and tumorsphere formation evaluation. The other 6 mice in each group were fed for survival recording over 60 days. Mice were euthanized when their body weight losses were >15% or when tumor volumes exceeded 2000 mm^3^.

### In Vivo Antitumor Efficacy of CALT‐GM‐NVs with Radiotherapy Against Subcutaneous HCC models—*Histology Analysis*

The tissues were fixed with 4% PFA, embedded in paraffin, and cut into slices with a 6 µm thickness. Pin1 and Notch1 immunohistochemistry analysis of tumor tissues was performed according to the manufacturer's instructions, and the slices were imaged by an optical microscope (Olympus BX51, Japan). TUNEL and H&E staining were performed according to the standard protocols of the corresponding assay kits. To detect the expression of Nanog and Oct4, tumor tissues were dehydrated in 30% sucrose, embedded with frozen O.C.T., and cut into frozen slices with 8 µm thickness, followed by immunofluorescence staining as described above.

### In Vivo Antitumor Efficacy of CALT‐GM‐NVs with Radiotherapy Against Subcutaneous HCC Models—*Evaluation of In Vivo CSC Differentiation*

The isolated tumors were immediately immersed in a pre‐chilled DMEM medium. The tumors were cut into small pieces and digested with a mixture of 1 mg mL^−1^ of Collagenase type IV and 1 U mL^−1^ of Dispase enzyme for 1.5 h at 37 °C. The tumor cells were filtered twice using a 70 µm filter to obtain single cells. Then, the appropriate amount of erythrocyte lysate was added to remove the erythrocytes and obtain the tumor cell suspension. To observe the tumor‐sphere‐forming ability of the tumor cells after different treatments, the isolated cells were seeded in 96‐well ultra‐low attachment plates (1000 cells per well) and cultured with the serum‐free CSC medium. The size of tumor spheroids was measured on day 7. The populations of intratumoral CSCs after various treatments were evaluated by flow cytometry analysis of CD133, ABCG2, and CD24 expressions.

### In Vivo Antitumor Efficacy of CALT‐GM‐NVs with Radiotherapy Against Orthotopic HCC Models

After orthotopic implantation of luciferase‐labeled MHCC97H cells, the mice were intraperitoneally injected with 100 mg kg^−1^ D‐luciferin 5 min before bioluminescent imaging. According to the bioluminescence signals, mice were divided into 3 groups (8 mice per group). The mice were injected via the tail vein with 100 µL of PBS or CALT‐GM‐NVs (equivalent ATRA and GSI concentrations of 10 and 8.7 µg mL^−1^, respectively). At 12 h post‐administration, mice were irradiated with 6 Gy of γ‐radiation. The changes in body weight were recorded over 15 days. Tracking in situ tumor changes by monitoring liver bioluminescence signals on days 0, 6, and 12. They were sacrificed on day 14, and the tumor tissues were collected for histology analysis.

### In Vivo Antitumor Efficacy of CALT‐GM‐NVs with Radiotherapy Against PDX Models

For the construction of CALT‐GM‐NVs, the cell membrane of human tumor tissue (hT‐NVs) was digested into a single‐cell suspension, and then CALT‐GM‐NVs were constructed according to the aforementioned HCC cells. For evaluation of the antitumor efficacy of CALT‐GM‐NVs, PDX mice (18 mice per group) were randomly in 3 groups when the tumor volume was ≈100 mm^3^. Mice were injected via the tail vein with 100 µL of PBS or CALT‐GM‐NVs (equivalent ATRA and GSI concentrations of 10 and 8.7 µg mL^−1^, respectively). At 12 h post‐administration, mice in the PBS+IR group and the CALT‐GM‐NVs+IR group received 6 Gy of γ‐radiation. On day 2 after treatment, 5 mice per group were executed, and their tumors and livers were collected for histological and stemness analysis. On day 9, when the tumor volume of the PBS group exceeded 2000 mm^3^, 5 mice in each group were executed, and the tumor tissues were photographed and weighed. On day 14, relapsed mice in the PBS+IR (*n* = 8) and CALT‐GM‐NVs+IR (*n* = 2) groups received the second treatment. Tumor volumes, recurrence, and body weights of 8 mice in each group were monitored daily over 28 days. The survival of these mice was recorded within 60 days.

### Statistical Analysis

For all data, the mean ± S.D. (standard deviation) was expressed. Statistical analysis was performed with GraphPad Prism 8.0.1 (GraphPad Software, Inc., San Diego, CA) software by two‐tailed unpaired Student's t‐tests, log‐rank test, or one‐way ANOVA. Differences between samples were considered statistically significant when *p* < 0.05. The sample size (*n*) for each statistical analysis was at least 3 (see details of various experimental methods and figure legends).

## Author Contributions

H.M.C. and Q.W. contributed equally to this work. J.F.L., L.Y., W.Y., and H.C. conceived and designed the study; H.C. and H.J. collected and analyzed the clinical samples; H.C. constructed the nanovesicles and performed in vitro experiments; H.C. and Q.W. performed CSCs sorting and in vitro culture experiments; Y.N. and H.C. performed in vivo experiments on PDX models; S.W., D.W., and J.J.L. helped with the animal experiments and facilitated the data and file processing; H.C. and L.Y. wrote the manuscript; L.Y., W.Y., and J.F.L. revised the manuscript. All authors discussed the results and commented on the manuscript.

## Conflict of Interest

The authors declare no conflict of interest.

## Supporting information



Supporting Information

## Data Availability

The data that support the findings of this study are available from the corresponding author upon reasonable request.
