# Supplementary material for: Dual‐Targeted Nanovesicles Induced Cancer Stem‐Like Cell Differentiation to Sensitize Hepatocellular Carcinoma Radiotherapy
Source: Adv Sci (Weinh). 2025 Jul 23;12(39):e02409. doi: 10.1002/advs.202502409 (PMC12533360; doi:10.1002/advs.202502409)
Supplement: Supplementary file 1 — Supporting Information [file ADVS-12-e02409-s001.docx]

**Dual-Targeted Nanovesicles Induced Cancer Stem-Like Cell Differentiation to Sensitize Hepatocellular Carcinoma Radiotherapy**

Hongmei Cao^1†^, Qian Wang^1†^, Yanan Niu^2^, Shuxiang Wang^1^, Haixue Jia^1^, Dianyu Wang^1^, Jinjian Liu^1^, Wei Yuan^2, *^, Lijun Yang^1, *^, Jianfeng Liu^1, *^

^1^State Key Laboratory of Advanced Medical Materials and Devices, Tianjin Key Laboratory of Radiation Medicine and Molecular Nuclear Medicine, Key Laboratory of Radiopharmacokinetics for Innovative Drugs, Tianjin Institutes of Health Science, Institute of Radiation Medicine, Chinese Academy of Medical Sciences & Peking Union Medical College, Tianjin 300192, P. R. China

^2^State Key Laboratory of Molecular Oncology, National Cancer Center/National Clinical Research Center for Cancer/Cancer Hospital, Chinese Academy of Medical Sciences and Peking Union Medical College, Beijing, 100021, People’s Republic of China

^†^These authors contributed equally to this work.

^*^Corresponding e-mail: liujianfeng@irm-cams.ac.cn; [yanglijun@irm-cams.ac.cn](mailto:yanglijun@irm-cams.ac.cn); yuanwei@cicams.ac.cn)

Supplementary materials and methods

**1. Reagents.**

NH_2_-PEG_2000_-DSPE, HSPC, and Cholesterol were purchased from AVT company (Shanghai, China). 2-Methylimidazole (2-MIM, 99%), Zinc nitrate hexahydrate (Zn (NO_3_)_2_·6H_2_O, 99%), ATRA, and 50 × B-27 supplement were purchased from Sigma-Aldrich. Insulin was bought from MedChemExpress (MCE). MK-0752 (GSI) was purchased from Selleck company. Epidermal growth factor (EGF), basic fibroblast growth factor (bFGF), DiO, DiI, DiR, and Actin-Tracker Red-Rhodamine were purchased from Beyotime Biotechnology. Crystal violet, DAPI, H&E staining kit, and TUNEL kit were obtained from Solarbio. APC/FITC/PE-conjugated anti-human CD133 (566593), FITC conjugated anti-human CD24 (555427), PE conjugated anti-human CD44 (555794), and apoptosis kit were purchased from BD company. FITC conjugated ABCG2 (332014) was purchased from BioLegend company. All experiments were performed with ultrapure water (Millipore Milli-Q grade).

**2. Cell culture, CSC sorting, and stemness identification.**

*2.1 Cell culture.* Human hepatocellular carcinoma (HepG2, catalog no. SCSP-510 and Bel7402, catalog no. NM-G04), human normal liver cell (LO2, catalog no. CC-Y1237), and mouse fibroblast cells (L929, catalog no. SCSP-5039) were obtained from the American Type Culture Collection (ATCC). The luciferase-transfected HCC cell lines (MHCC97H, catalog no. CC-Y1613) were purchased from FuHeng Biology (Shanghai, China). HepG2, MHCC97H, and LO2 cells were cultured in DMEM (Gibco, Grand Island, NY) medium containing 10% fetal bovine serum (FBS, Gibco) and 1% Penicillin Streptomycin (P/S, Gibco) under 5% CO_2_ and 95% humidified air at 37 °C. Bel7402 and L929 cells were cultured in RPMI-1640 medium with all recommended supplements. Mouse bone marrow-derived stem cells (BM-MSCs) were isolated from the femurs and tibias of 6–8-week-old C57BL/6 mice. Briefly, the bone marrow was flushed out using sterile PBS supplemented with 2% FBS and passed through a 70 μm cell strainer. After centrifugation, cells were resuspended and cultured in RPMI-1640 medium with all recommended supplements. The medium was changed every 2–3 days, and cells at passages 2–4 were used for subsequent experiments.

*2.2 CSCs sorting and culture.* To obtain CSCs, a flow sorting method was used to isolate and enrich CD133^+^ cell subpopulations. Before cell sorting, parental cells were precultured with serum-free medium for 24 h. When cell fusion reached approximately 80%, HepG2, Bel7402, and MHCC97H cells (1 × 10^7^) were suspended in PBS and then incubated with APC-conjugated anti-CD133 antibody for 30 min at 4 °C. Cells were washed in PBS and resuspended at 1 × 10^7^ cells in PBS and filtered with a 70 μm strainer. Then, the CD133^+^ cells were sorted by flow cytometry (FACS Calibur; BD Biosciences, San Jose, CA). The sorted CD133^+^ cells were cultured in 3D spheroid formation with serum-free CSC medium containing DMEM/F-12, 1×B-27 supplement, EGF (20 ng/mL), bFGF (20 ng/mL), insulin (5 μg/mL), and 1% of P/S. For *in vitro* propagation, CSC tumorspheres were collected and separated into single cells by 0.25% trypsin digestion, centrifuged at 600 g for 5 min to collect the single cell suspension, and then inoculated in an ultra-low attachment 6-well plate or 96-well Nunclon Sphera plates (Thermo Scientific) for further experiments.

*2.3 Stemness identification.* The stemness-related property of CSC tumorspheres was identified by evaluating radiotherapy resistance, self-renewal ability, and the expression of stemness-related factors (Nanog, Oct4, and Sox2) and CSC markers (CD133).

**3. Synthesis and characterization of CALT-GM-NVs.**

*3.1 Synthesis of GM.* First, 200 mg (2.436 mmol) of 2-MIM was dissolved in 1 mL of methanol, and 1 mg of GSI was added and stirred for 5 min. 20 mg (0.066 mmol) of Zn (NO_3_)_2_-6H_2_O was then dissolved in 80 μL of methanol and slowly added dropwise, and the solution was observed to turn milky white. The product was collected by centrifugation at 10,000 rpm and washed at least three times with methanol. The product was dried under vacuum at 37 °C for 2 h to obtain the powder.

*3.2 Preparation of T-NVs.* T-NVs were harvested as previously reported. Briefly, HepG2, Bel7402, or MHCC97H cells were suspended at a concentration of 2 × 10^7^ cells/mL in a precooled hypoosmotic buffer containing 1x protease inhibitors (pH 7.4, 10 mM of Tris, 1 mM of MgCl_2_), ice lysed for 30 min, then the cell suspension liquid nitrogen repeated freeze-thaw 5–10 times lysis cells. The cell lysate was collected in a centrifuge tube at 2000 rpm and centrifuged at 4 °C for 10 min. The supernatant was collected in a new centrifuge tube at 14,000 rpm and centrifuged at 4 °C for 30 min to obtain membrane precipitate. Subsequently, the membrane was resuspended in sterile water containing protease inhibitors, and membrane fragments were obtained by sonication.

*3.3 Synthesis and characterization of CP-PEG_2000_-DSPE.* The CP peptide was synthesized using the Fmoc solid-phase synthesis method that we reported previously, and purified by high-performance liquid chromatography (HPLC), dried by lyophilization, and confirmed by mass spectrometry. CP-PEG_2000_-DSPE was obtained after the reaction between NH_2_-PEG_2000_-DSPE and CP peptides. Briefly, 40 mg of CP peptide was dissolved in 3 mL of DMF. Then, EDC and n-hydroxysuccinimide (NHS) (CP: EDC: NHS = 1:5:10, molar ratio) were added, and the mixture was stirred in an ice bath for 2 h to activate the carboxyl groups on the CP peptide. To remove the protecting group from the CP peptide, 10 mL of TFA (95%) was added, followed by 30 min of stirring. The pH was adjusted to 7.4 with NaOH (3 M), and the crude product was obtained by rotary evaporation. After being dialyzed with pure water for 48 h and lyophilized, pure CP-PEG_2000_-DSPE was obtained and confirmed by time-of-flight mass spectrometry (TOF-MS).

*3.4 Preparation of CALT-GM-NVs.* Liposomes containing ATRA, DiO, DiI, or DiR were prepared by thin-film hydration. As an example of containing ATRA liposomes synthesizing, HSPC, cholesterol, CP-PEG_2000_-DSPE, and ATRA were dissolved in 1.37 mL of chloroform at a lipid concentration of 10 mg/mL in a molar ratio of 2:10:1:1. The film was then evaporated into a dry film using a rotary evaporator at 55 °C in a round bottom flask. Then, 4 mL of sterile water was added to make the lipid concentration 2.5 mg/mL, and the lipid film was hydrated by sonication in a water bath at 55 °C. The lipid membrane fragments were further sonicated in an ice bath (100 W, 2 min) to obtain lipid membrane fragments. To obtain CALT-NVs, CAL-NVs (1 mg/mL) were vortexed and sonicated with 100 μg of T-NVs (protein concentration) in an ice bath for 5 min and dialyzed in a 3500 Da dialysis bag for 12 h to remove the unencapsulated ATRA. To obtain CALT-GM-NVs, 1 mL of CALT-NVs (1 mg/mL) was mixed with 2 mL of GM (0.5 mg/mL) in deionized water. The mixed suspension was sonicated for 10 min and stirred at 600 rpm for 30 min until clarified and clarified. Finally, the obtained CALT-GM-NVs were purified by centrifugation at 10000 rpm for 5 min to remove the free membrane.

**4. Characterization of the CALT-GM-NVs.**

*4.1 Analysis the surface morphology and size of different nanovesicles.* T-NVs, GM, CAL-NVs, CLT-GM-NVs, ALT-GM-NVs, and CALT-GM-NVs were observed by TEM (HITACHI-HT7800). Drop 10 μL of each sample (1 mg/mL) onto the copper wire with carbon film and let stand for 2 min, followed by blotting the excess sample with filter paper. Then, the T-NVs and biomimetic nanovesicles were negatively stained with 1% of uranyl acetate for 90 s, and the excess solution was dried with filter paper. After drying at room temperature, the samples were detected by TEM. The hydrodynamic diameter and surface charge of different nanovesicles were analyzed using a zetasizer (Brookhaven). Powder XRD mapping using a Panalytical X Pert Pro X-ray powder diffractometer with Cu Kα radiation (40 V, 40 mA, λ = 1.54056 Å) in the θ-θ mode from 20° to 90° (2 θ). Standard deviations were calculated with 3 runs. For the stability study, GM and CALT-GM-NVs were stored at 4 °C in PBS or PBS containing 10% of FBS, and hydrodynamic diameter was measured periodically by a zetasizer.

*4.2 Drug loading and in vitro drug release study.* To determine the contents of ATRA and GSI in the biomimetic nanovesicles, the freshly prepared nanovesicles were vortexed in 10 mL of PBS (pH 5.5) containing 10% Triton X-100 for 1 h at room temperature. Then the solution was centrifuged at 14000 g for 30 min, and the contents of ATRA and GSI in the supernatant were determined by UV-Vis spectrophotometer (TU-1801) at 350 nm and 273 nm, respectively. The DLE and DLC were calculated as follows:

DLE (%) = mass of drug encapsulated in nanovesicles /

initial mass of drug × 100

DLC (%) = mass of drug encapsulated in nanovesicles /

total mass of nanovesicles × 100

For drug release analysis, 1 mL of CALT-GM-NVs suspensions was dialyzed in a dialysis bag (molecular weight cutoff of 1000 Da) in 20 mL of PBS (pH 5.5 or 7.4) with or without 1% Triton X-100. At different time intervals, 1 mL of the dialysis buffer was collected for ATRA and GSI quantification, and another 1 mL of PBS (same pH) was added into the system. Released ATRA and GSI were quantified by determining the absorbance at 350 nm and 273 nm by UV–vis spectrometry.

*4.3 Verification of the fusion of T-NVs and CAL-NVs.* To check whether liposomes were incorporated into T-NVs, T-NVs were incubated with PKH26 (excitation/emission = 551/567 nm) for 15 min on ice. The mixture was then centrifuged at 14,000 g for 30 min at 4 °C to remove the excess dye. Then, the DiO (excitation/emission = 484/501 nm)-labeled CAL-NVs and PKH26-labeled T-NVs were fragmented by ultrasound and hydration to form hybrid nanovesicles, which were detected by the colocalization of CAL-NVs and T-NVs under a CLSM (Nikon, C2).

**5. Cell cytotoxicity study.**

Study the cytotoxicity of the biomimetic nanovesicles by using the cell counting kit-8 (Cck-8, BestBio, China). Briefly, HepG2, LO2, BM-MSCs, and L929 cells were seeded at 4 × 10^3^ cells per well on a 96-well plate. After 12 h of seeding, with different ATRA (0, 1.25, 2.5, 4.0, 5.0, and 10.0 μg/mL) and GSI concentrations (0, 1.08, 2.17, 3.48, 4.35, and 8.75 μg/mL) of biomimetic nanovesicles were added for another 72 h. Then, each well was incubated with 10 μL of CCK-8 in an FBS-free medium for 2–4 h, and the absorbance at 450 nm was measured with a MicroplateReader (Thermo Varioskan Flash).

**6. Calculation of the combination index**

CSC-enriched cells were inoculated in a 96-well plate (5000 cells/well). Then the cells were incubated with ATRA, GSI, or their combination for 48 h and radiated at 6 Gy. Cell viability was measured using CCK-8 assays. The half-maximal inhibitory concentration (IC₅₀, μg/mL) and combination index (CI₅₀) were calculated based on the Chou–Talalay method using CompuSyn software. CI₅₀ was calculated as: CI₅₀ = (D)₁/(Dx)₁ + (D)₂/(Dx)₂. where (D)₁ and (D)₂ are the concentrations of ATRA and GSI in combination to achieve 50% inhibition, and (Dx)₁ and (Dx)₂ are the respective concentrations used alone to reach the same effect. A CI₅₀ value <1 indicates synergy, =1 additive effect, and >1 antagonism.

**7. Evaluate the ability of CALT-GM-NVs to inhibit Pin1 and Notch1.**

The expression of Pin1, Notch1, and their target genes was detected by Western blot. Briefly, 100000 cells/well of CSCs were inoculated into 6-well ultra-low attachment plates for 72 h and continued co-incubated with biomimetic nanovesicles (equivalent ATRA and GSI concentration of 5 µg/mL and 4.35 µg/mL, respectively) for another 48 h. The cells were lysed to obtain protein and incubated with anti-human antibodies (Pin1, cleaved Notch1, HES-1 (1:1000, GeneTex, GTX108356, USA), CDK1 (1:1000, Abcam), AKT (1:1000, Abcam), β-catenin (1:1000, Abcam), and β-actin (1:1000, Abcam)) overnight at 4 °C as above mentioned.

For Pin1 and HES-1 mRNA levels analysis, the RNA samples were extracted from treated cells according to the manufacturer’s protocols. The gene was expressed as 2-(^△△^CT) after normalization by *GAPDH* as described above.

For Pin1 and Notch1 immunofluorescence co-staining, 1000 cells/well of CSCs were inoculated in 96-well Nunclon Spheres plates for 72 h and continued co-incubated with biomimetic nanovesicles (equivalent ATRA and GSI concentration of 5 µg/mL and 4.35 µg/mL, respectively) for another 48 h. Then, tumorspheres were collected and fixed with 4% PFA for 10 min. After PBS washing, the cells were blocked with 5% of bovine serum albumin (BSA) for 1 h at room temperature and then incubated with anti-human antibodies Pin1 (1:200; Abcam) and Notch1 (1:200; Abcam) overnight at 4 °C. After PBS washing, the cells were incubated with anti-rabbit AF488-IgG antibody (1:200; Abcam) and anti-mouse AF488-IgG or AF594-IgG (1:200; Abcam) for 1 h at room temperature. Finally, the cells were incubated with DAPI-negative staining of cell nuclei and observed under CLSM.

**8*. In vivo* safety analysis.**

*7.1 Hemolysis assay.* For hemolysis analysis, rat red blood cells were incubated at 37 °C for 2 h with different concentrations of nanovesicles (0.1, 0.2, 0.4, 0.6, 0.8, 1, and 10 mg/mL), PBS (negative control), and 0.1% Triton X-100 (positive control). After centrifugation, the absorbance was measured at 570 nm with a Microplate Reader.

*7.2 Blood routine and blood biochemical examination.* To evaluate the clinical potential of biomimetic nanovesicles, BALB/c mice (6–8 weeks, female) were randomly divided into 5 groups (3 mice per group), followed by intravenous administration with PBS (100 μL) or different biomimetic nanovesicles (equivalent ATRA and GSI concentration of 10 µg/mL and 8.7 µg/mL, respectively) two times at the interval of one week. Blood samples were then collected for blood chemistry assay and blood routine assay on day 14.

**9. Tumor models.**

*8.1 HCC cell line-derived subcutaneous tumor models.* 6–8 weeks Balb/c nude mice were purchased from HFK Bio-Technology (Beijing, China). 1 × 10^7^ HepG2 cells were administered subcutaneously to the right side of the mice. The mice were subjected to different treatments when the tumor volume reached about 100 mm^3^.

*8.2 HCC cell line-derived orthotopic tumor models*. 1 × 10^6^ luciferase-labeled MHCC97H cells were injected into the left lobes of the livers of 8-week-old Balb/c nude mice. The growth of liver orthotopic tumor cells was monitored by bioluminescent imaging (IVISTM 100 Imaging System, Xenogen).

*8.3 HCC patient-derived xenograft (PDX) tumor models.* A primary tumor sample was excised from an HCC patient and then subcutaneously inoculated into the axilla of 4–6-week-old male NOD.CB17-*Prkdc^scid^Il2rg^tm1^*/Bcgen mice that were purchased from Biocytogen (Beijing, China). After tumor formation, the tumor tissues were divided into two parts. One part of the tumor tissues was transplanted to Balb/c nude mice, and the third generation was used for experiments. Another part of the tissues was digested into a single cell suspension to isolate the membrane and construct hCALT-GM-NVS according to the previously mentioned method.

**10. Statistical analysis**

For all data, the mean ± S.D. (standard deviation) was expressed. Statistical analysis was performed with GraphPad Prism 8.0.1 (GraphPad Software, Inc., San Diego, CA) software by two-tailed unpaired Student’s t-tests, log-rank test, or one-way ANOVA. Differences between samples were considered statistically significant when *P* < 0.05. The sample size (n) for each statistical analysis was at least 3 (see details of various experimental methods and figure legends).

Supplementary Data


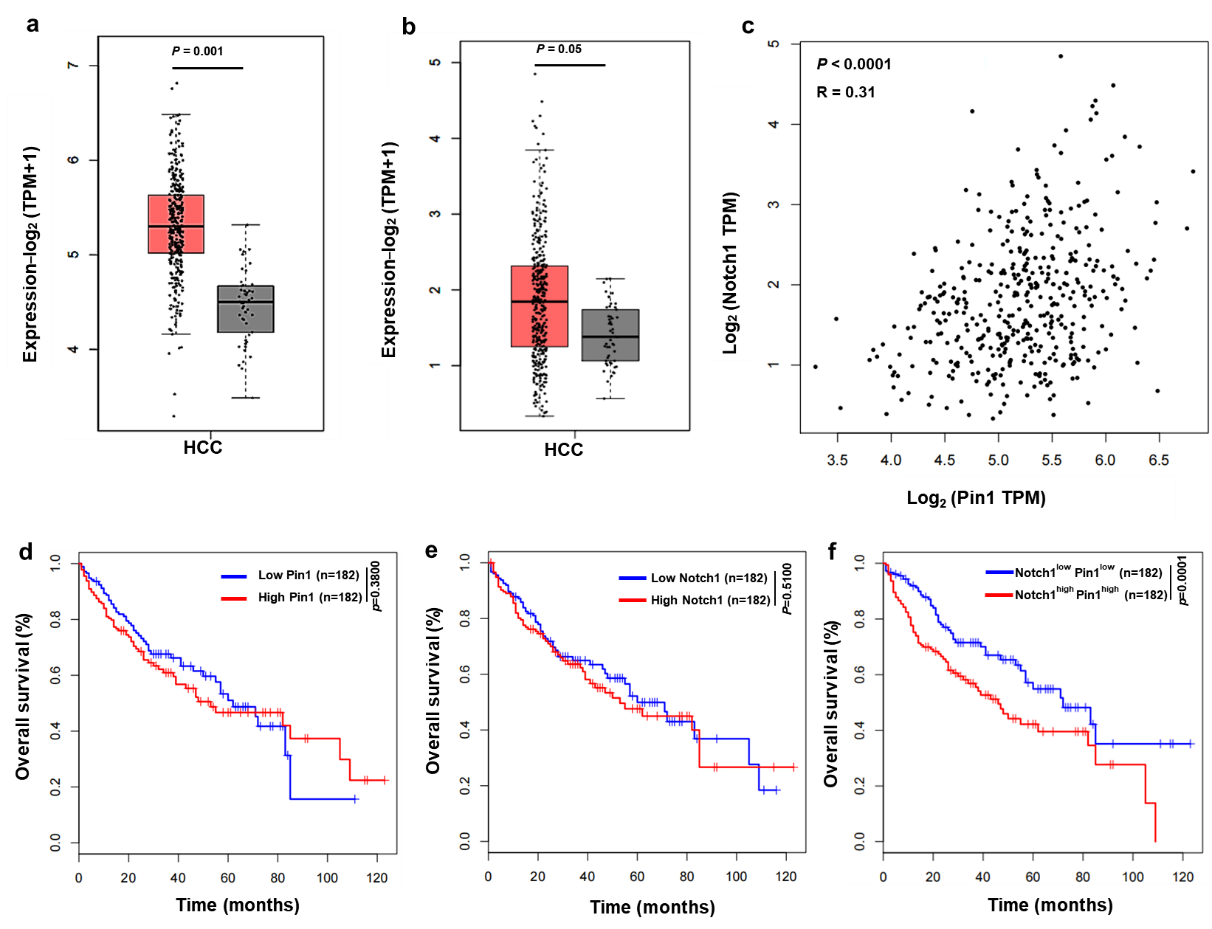


**Supplementary Figure 1.** A boxplot of Pin1 (**a**) and Notch1 (**b**) expression in HCC patients (*n* = 369) and healthy people (*n* = 50) screened from TCGA and Genotype-Tissue Expression (GTEx), determined by Gene Expression Profiling Interactive Analysis. The data were transformed as log2 (TPM + 1) and *n* is the number of biologically independent samples. **c** Correlative analysis of Pin1 and Notch1 expression in HCC patients. Survival curves of HCC patients with high (*n* = 182) or low (*n* = 182) Pin1 (**d**) and Notch1 (**e**) expression. **f** Survival curves of HCC patients with low Notch1 and Pin1 expressions (Notch1^low^ Pin1^low^, *n* = 182), and high Notch1 and Pin1 expressions (Notch1^high^ Pin1^high^, *n* = 182). The survival analyses were performed by the log-rank (Mantel–Cox) test.

**
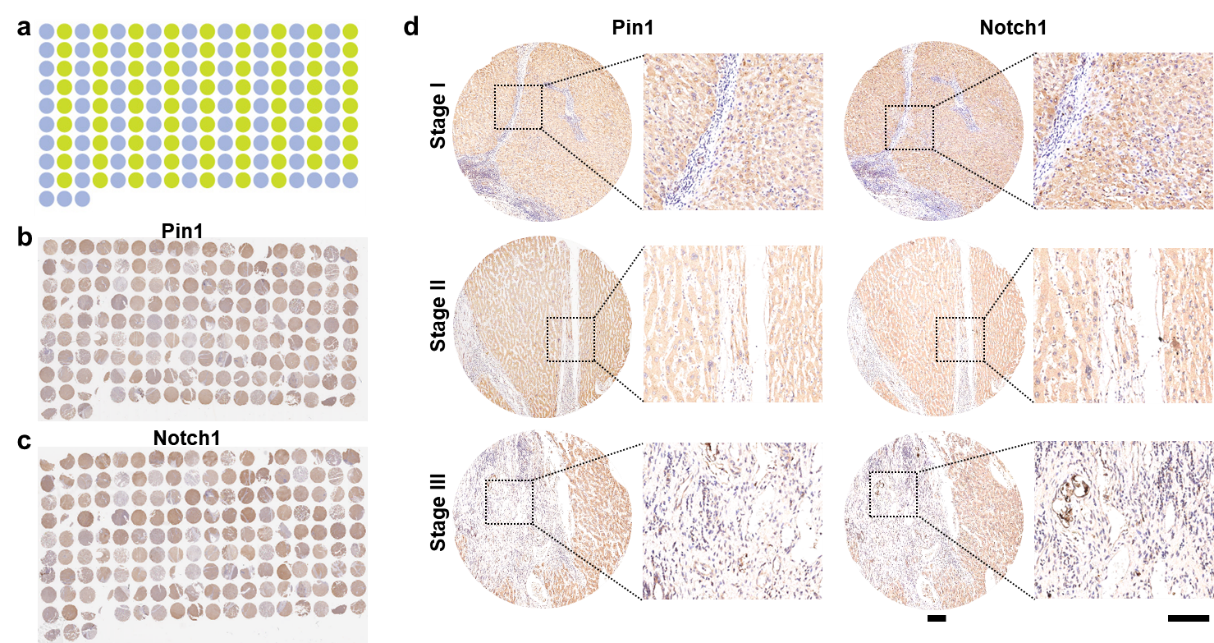
**

**Supplementary Figure 2. a** A tissue microarray (TMA) map consisting of HCC tissues (yellow) and the corresponding adjacent non-tumor liver tissues (blue). Pin1 (**b**) and Notch1 (**c**) immunohistochemistry staining of the clinical HCC tissues and the adjacent noncancerous tissues. **d** Representative images of the Pin1 and Notch1 expressions in adjacent noncancerous tissues (corresponding to Fig. 2b) at different HCC stages. Scale bar: 100 μm.


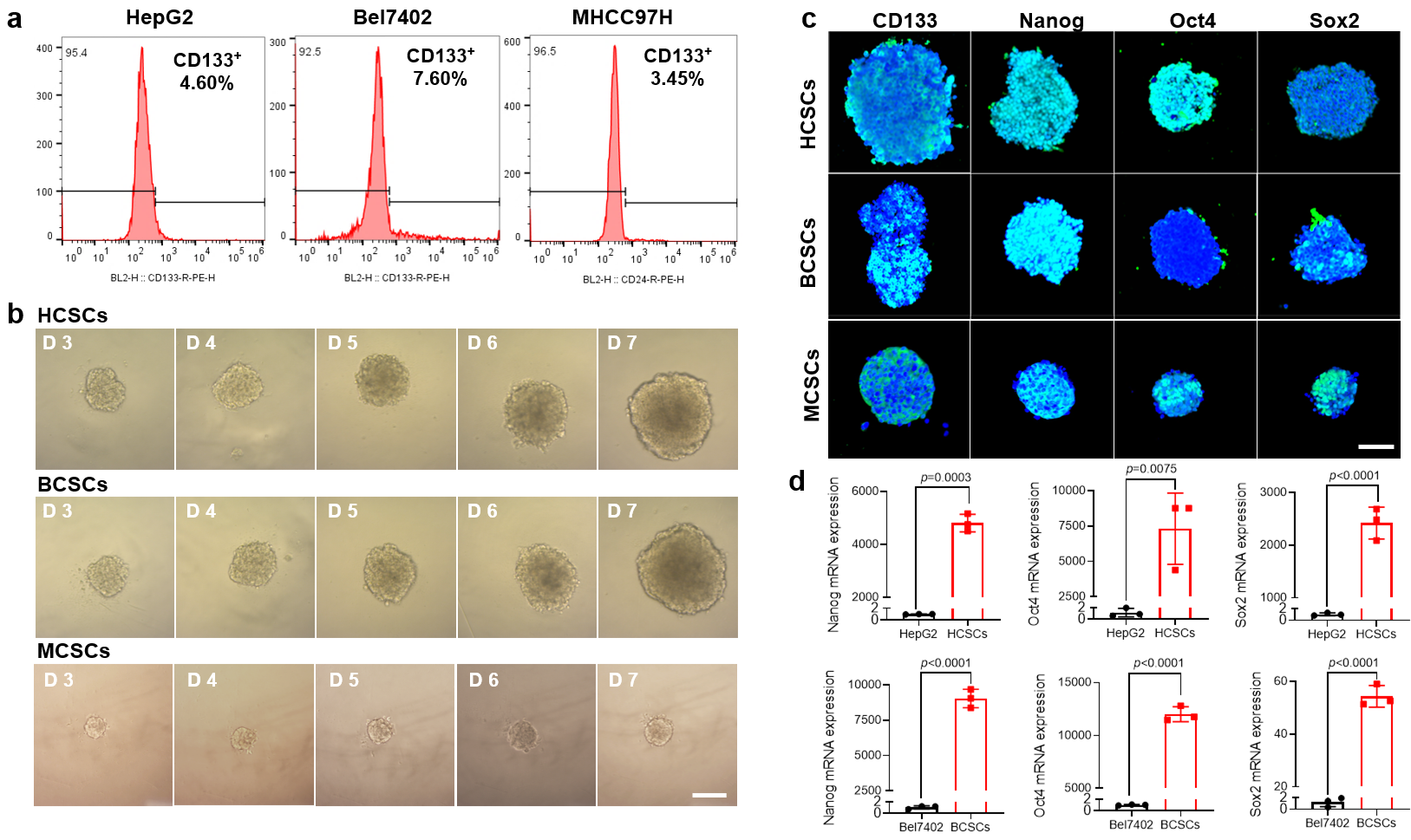


**Supplementary Figure 3. a** Representative images of flow cytometric sorting of CD133^+^ populations in HepG2, Bel7402, and MHCC97H cells. **b** Representative images of the tumor spheres formed by CD133^+^ populations cultured in the ultra-low attachment 96-well plates for 7 days. Scale bar: 100 μm. **c** Representative images of stemness-related protein expressions in tumor spheres. **d** Quantitative real-time PCR analysis of stemness-related gene expressions in CD133^+^ populations relative to their corresponding tumor cells (*n* = 3). Scale bar: 100 μm*. P* values were calculated by using a two-tailed unpaired Student’s *t*-test.


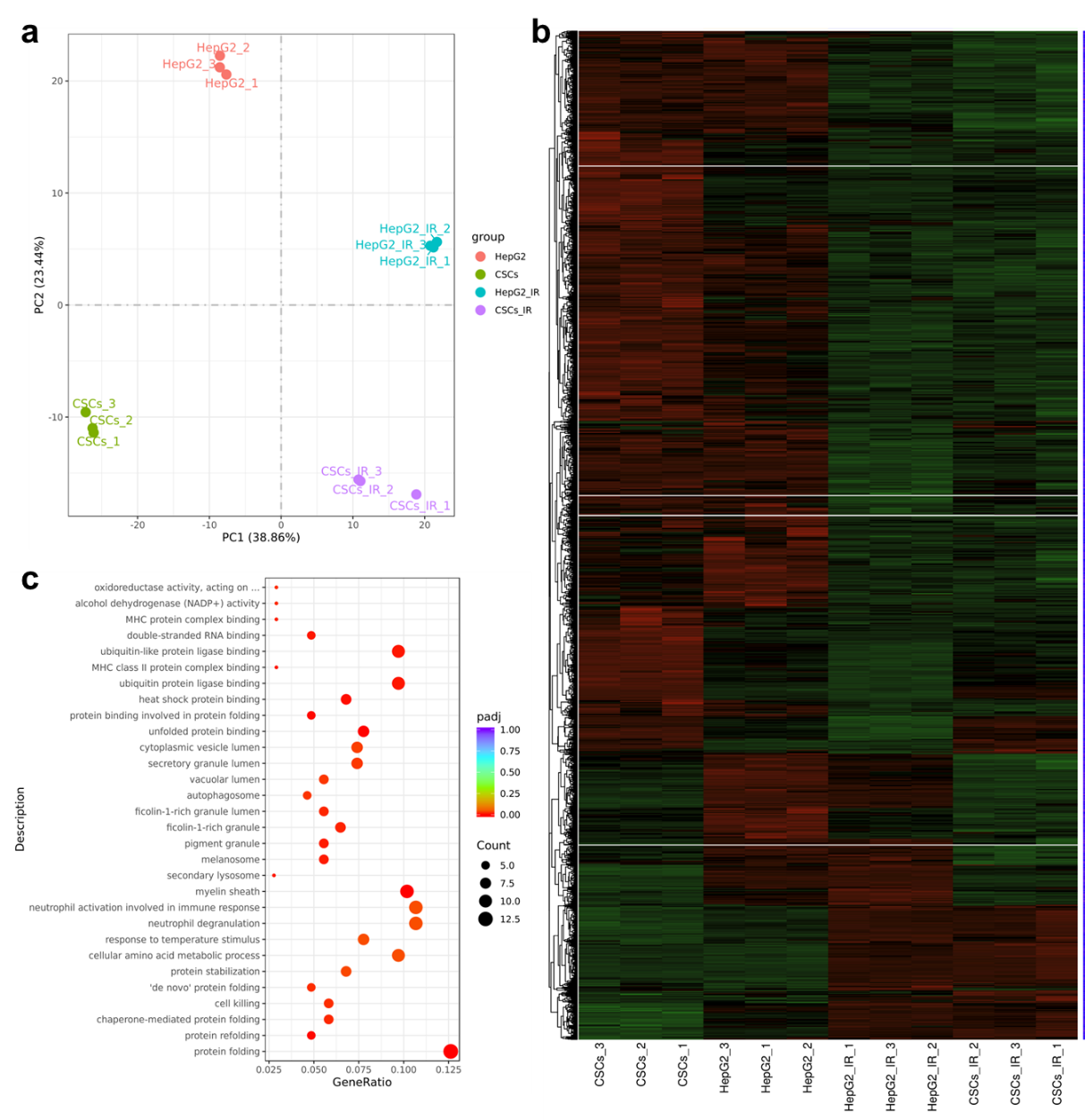


**Supplementary Figure 4. a** Principal component analysis of the entire transcriptomic profile of the HepG2, HepG2+IR, CSCs, and CSCs+IR. **b** Heat map of clustering analysis of differential genes between HepG2, HepG2+IR, CSCs, and CSCs+IR. **c** GO pathway enrichment in HepG2s+IR when compared to HepG2 cells.

**
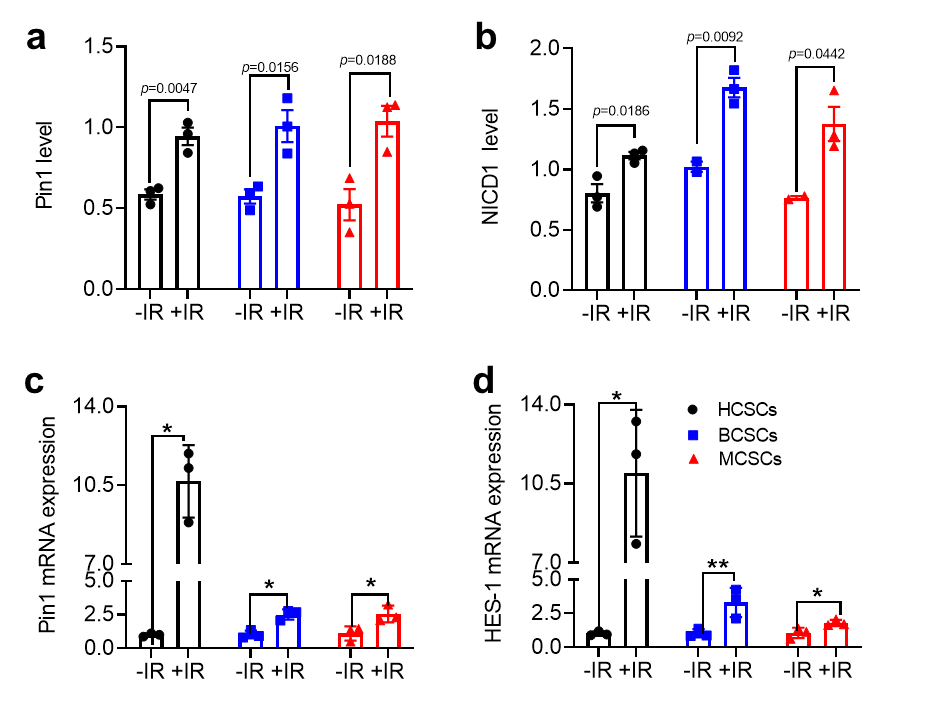
**

**Supplementary Figure 5.** Quantitative analysis of Pin1 (**a**) and NICD1 (**b**) protein levels in HCSCs, BCSCs, and MCSCs before and after 6 Gy of radiation. The relative mRNA level of Pin1 (**c**) and HES-1 (**d**) in CSCs after 6 Gy of radiation (n = 3). *P* values were calculated by using a two-tailed unpaired Student’s *t*-test.


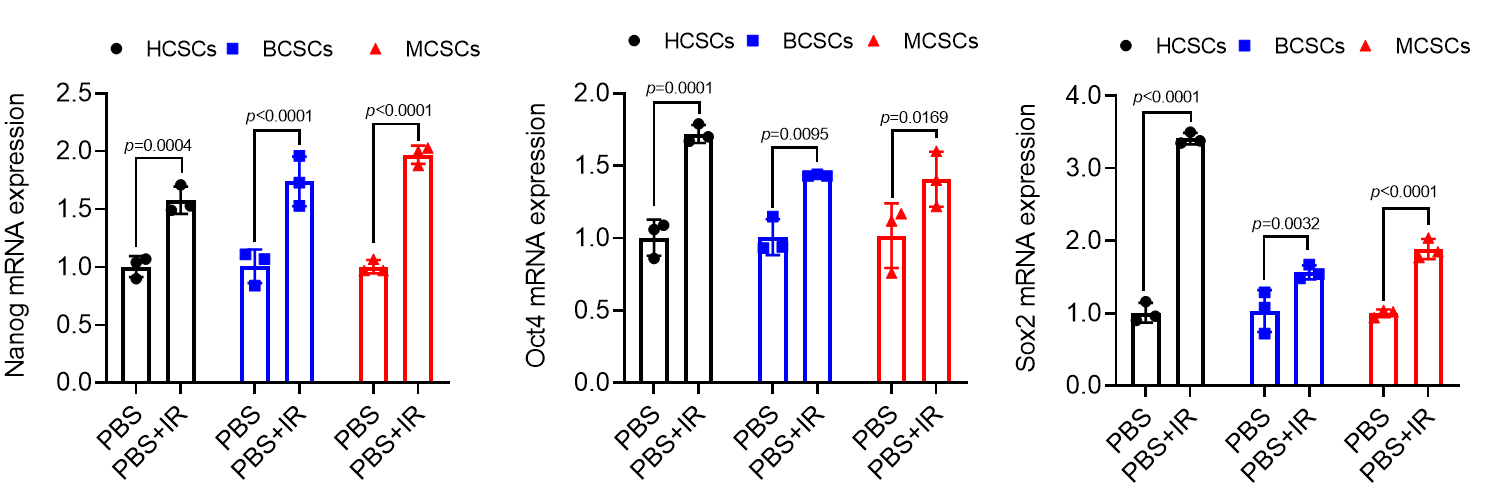


**Supplementary Figure 6.** The relative mRNA level of *Nanog*, *Oc4,* and *Sox2* in CSCs of three HCC cell lines after 6 Gy of radiation (*n* = 3). *P* values were calculated by using a two-tailed unpaired Student’s *t*-test.

**
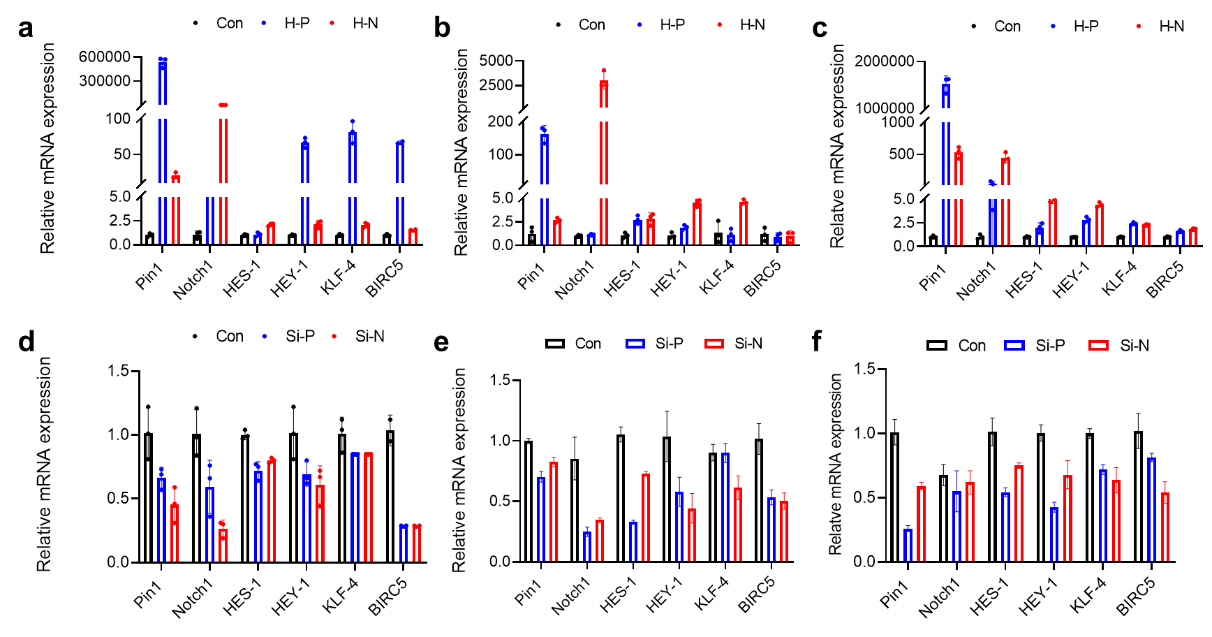
**

**Supplementary Figure 7.** Relative mRNA expression levels of Notch1, Pin1, and downstream Notch1 target genes (*HES-1*, *HEY-1*, *KLF-4*, *BIRC5*) were analyzed by qRT-PCR in HepG2 (**a**), Bel7402 (**b**), and MHCC97H (**c**) cells overexpressing Pin1 or Notch1 (H-P: Pin1-high; H-N: Notch1-high), and in HepG2 (**d**), Bel7402 (**e**), and MHCC97H (**f**) cells with Pin1 or Notch1 knockdown (Si-P: Pin1-silenced; Si-N: Notch1-silenced). Data represent mean ± SD (n = 3).

**
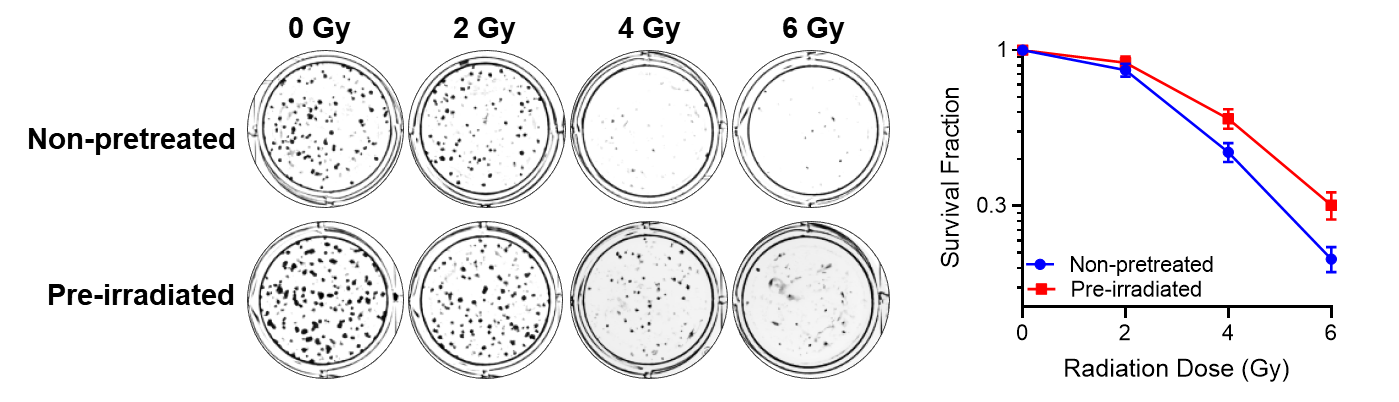
**

**Supplementary Figure 8.** Colony formation and clonogenic survival curves of primary HCC cells with or without prior irradiation, after subsequent treatment with 0, 2, 4, or 6 Gy radiation (*n* = 6).

**
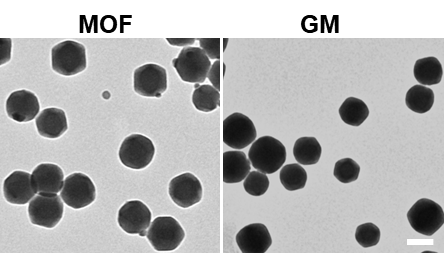
**

**Supplementary Figure 9.** Representative TEM images of MOF and GM. Scale bar: 100 nm.

**
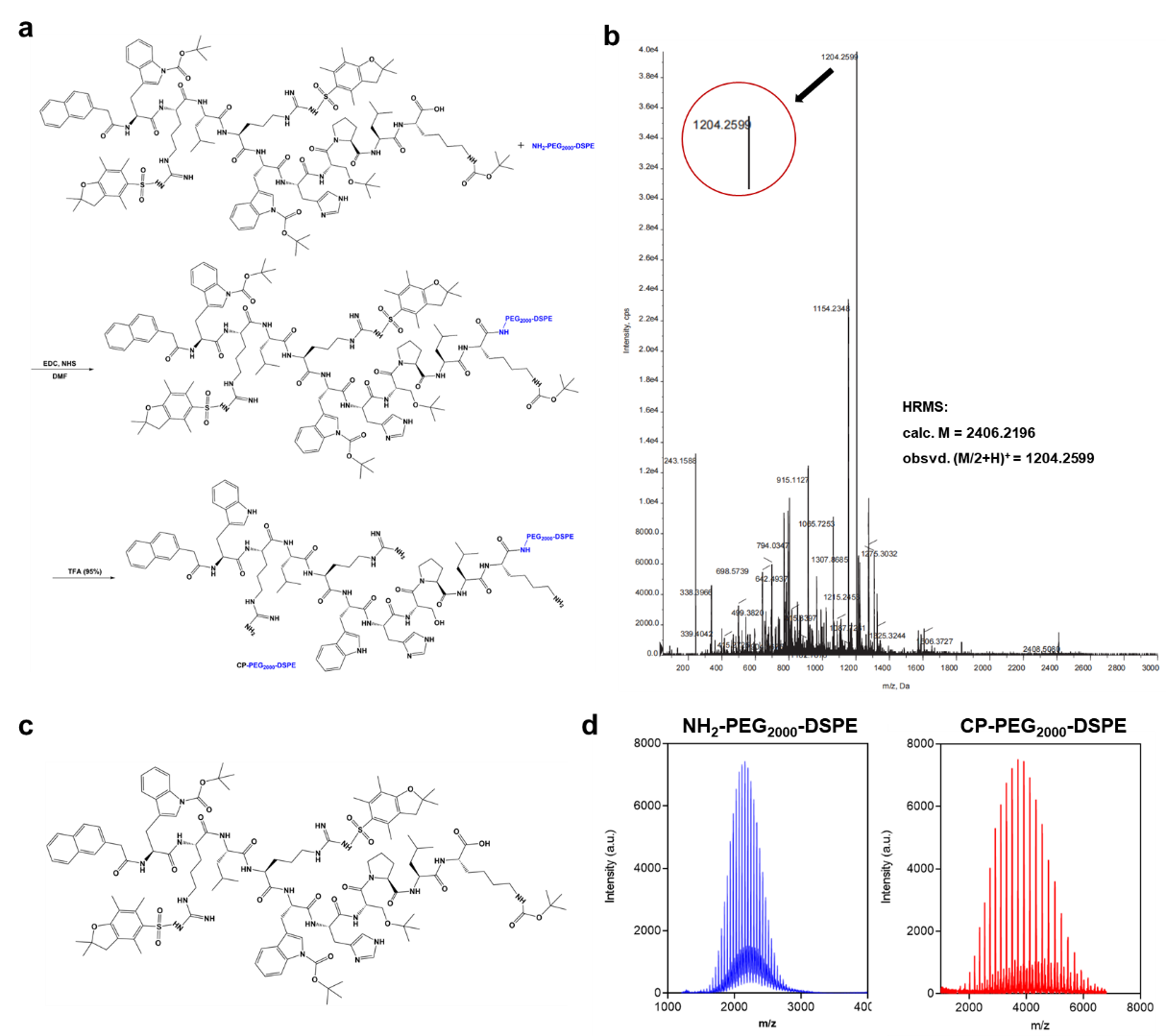
**

**Supplementary Figure 1****0.** **a** The synthesis route of CP-PEG_2000_-DSPE. **b, c** The HRMS spectrum of CP peptide with the side chain protecting groups. d TOF-MS spectra of NH_2_-PEG_2000_-DSPE and CP-PEG_2000_-DSPE.


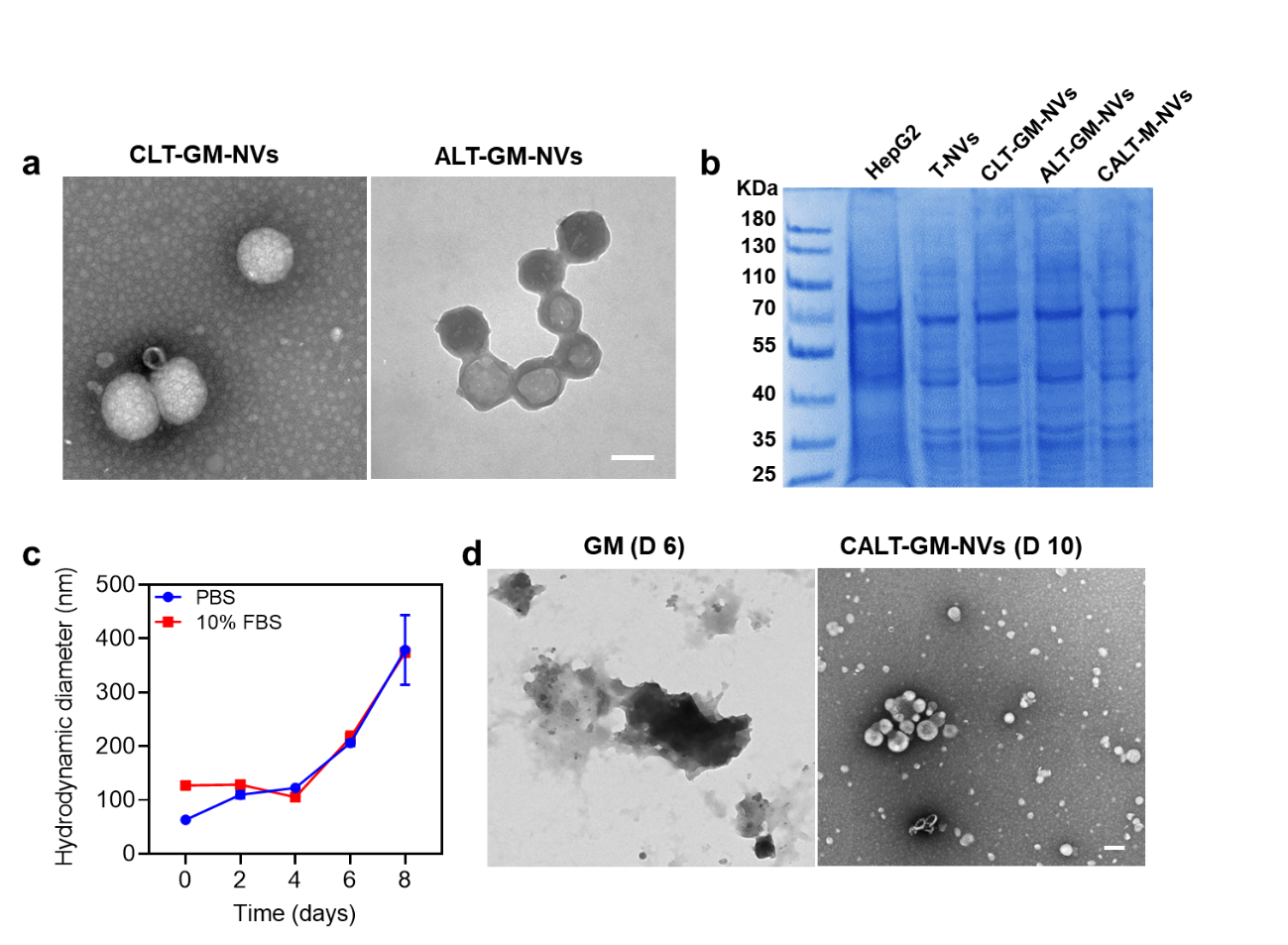


**Supplementary Figure 11. a** Representative TEM images of CLT-GM-NVs and ALT-GM-NVs. Scale bar: 100 nm. **b** SDS-PAGE analysis of protein distributions in HepG2, T-NVs, CLT-GM-NVs, ALT-GM-NVs, and CALT-GM-NVs. **c** Stability of GM over time in PBS with or without 10% of FBS (*n* = 3). **d** Representative TEM images of GM and CALT-GM-NVs in sterile water (pH 7.4) on days 6 and 10, respectively. Scale bar: 200 nm.


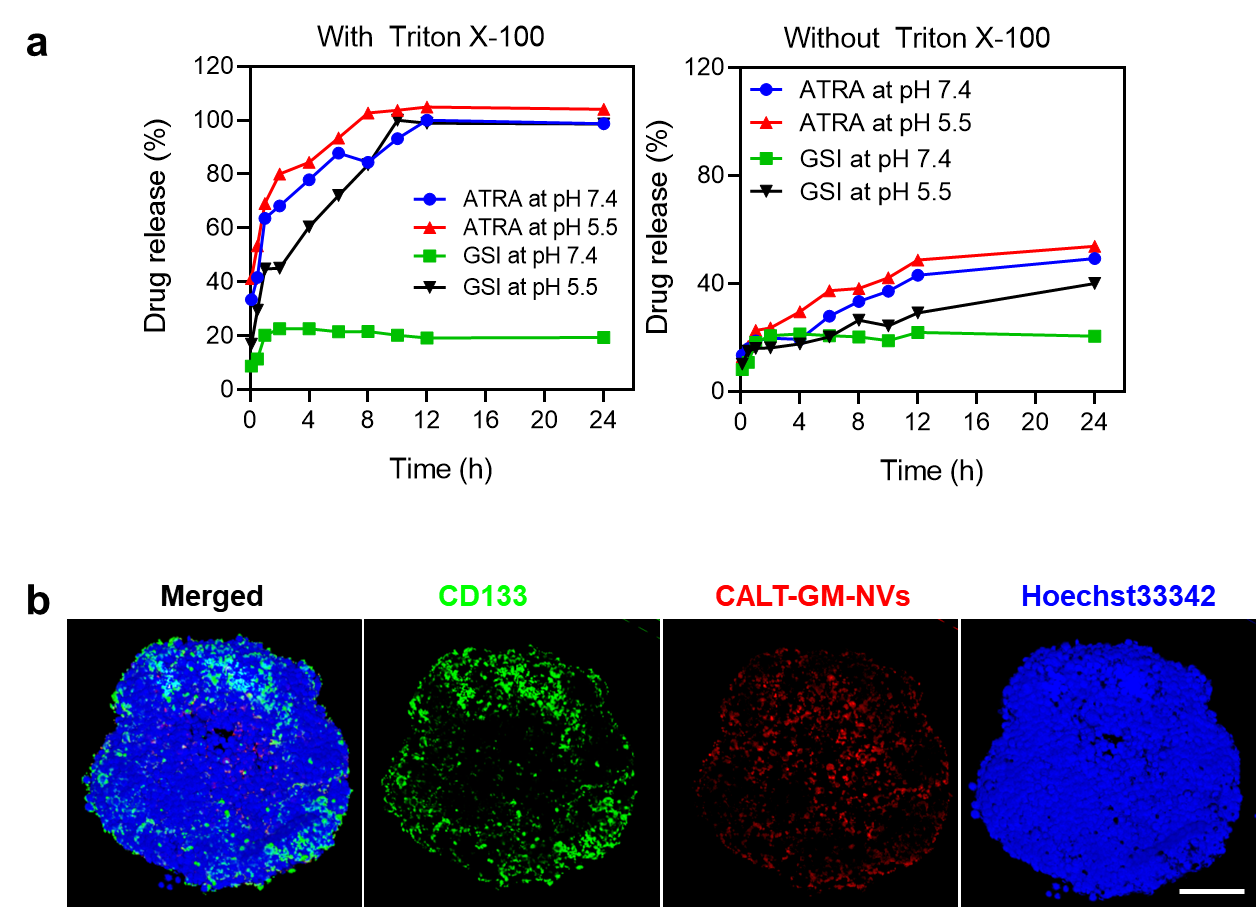


**Supplementary Figure 12. a** Drug release behaviors of CALT-GM-NVs in PBS (pH 5.5 or 7.4) with (h) or without (i) Triton X-100. **b** Colocalization of the DiI-labeled CALT-GM-NVs (red) and FITC-labeled CD133 antibody (green) in tumor spheres (DAPI: blue). Scale bar: 100 μm.

**
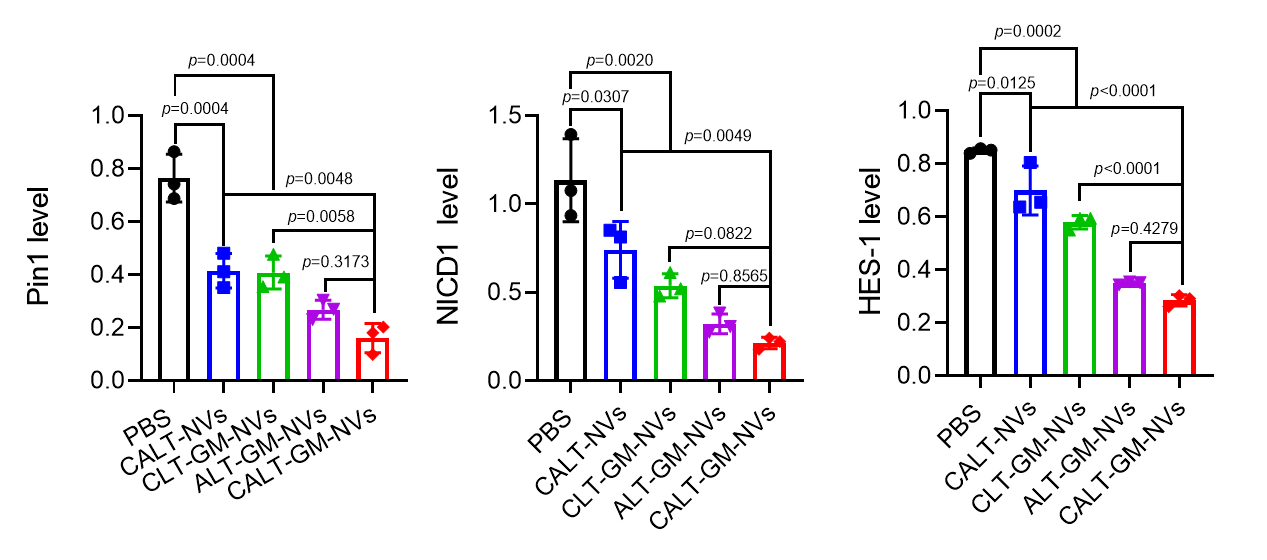
**

**Supplementary Figure 13**. Quantitative analysis of Pin1, NICD1, and HES-1 proteins in HCSCs after indicated treatments (*n* = 3). *P* values were calculated by using one-way ANOVA.


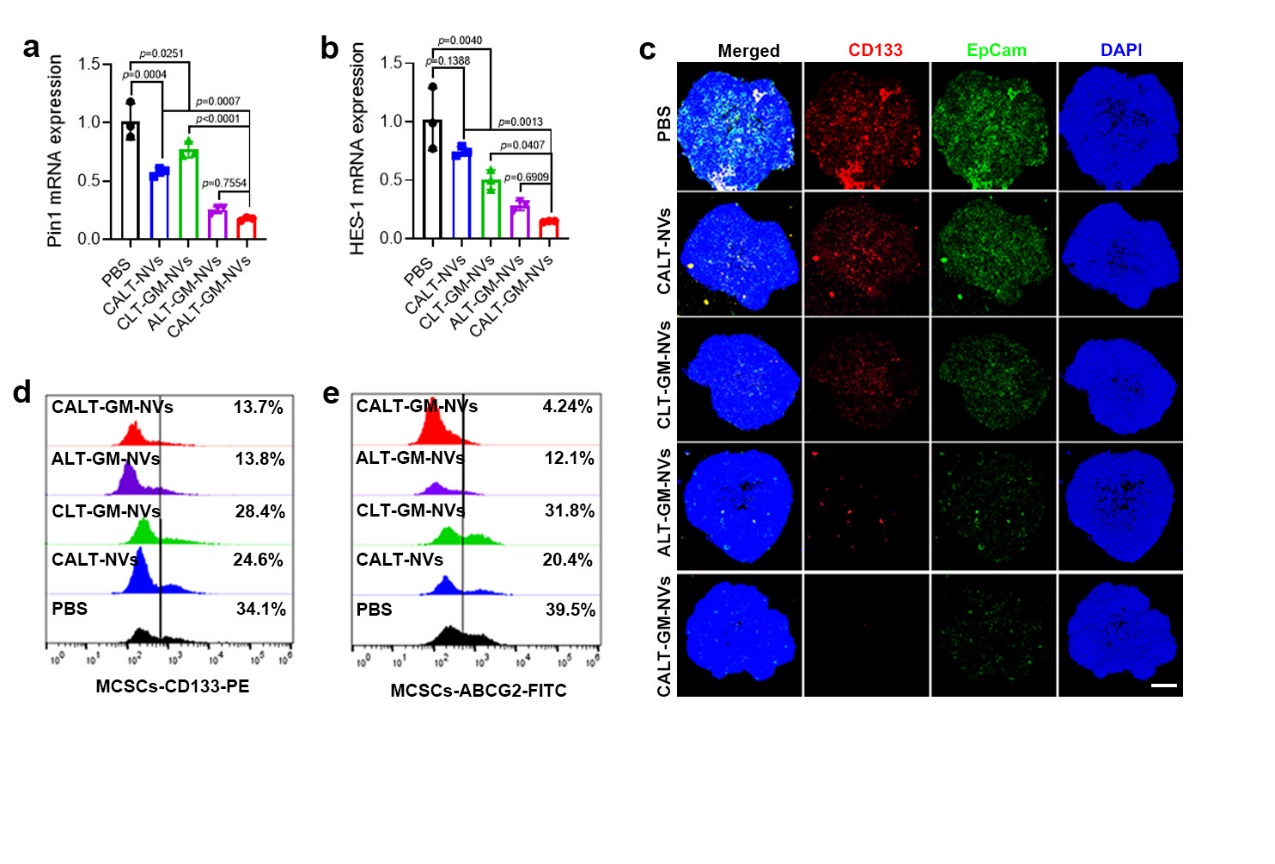


**Supplementary Figure 14**. The relative mRNA level of *Pin1* (**a**) and *HES-1* (**b**) in MCSCs after various treatments (*n* = 3). **c** Representative CLSM images of the Pin1 (green) and Notch1 (red) expression in MCSC tumorspheres after different treatments (DAPI: blue). The percentage of CD133^+^ (**d**) and ABCG2^+^ (**e**) populations in MCSCs was determined by flow cytometry.

**
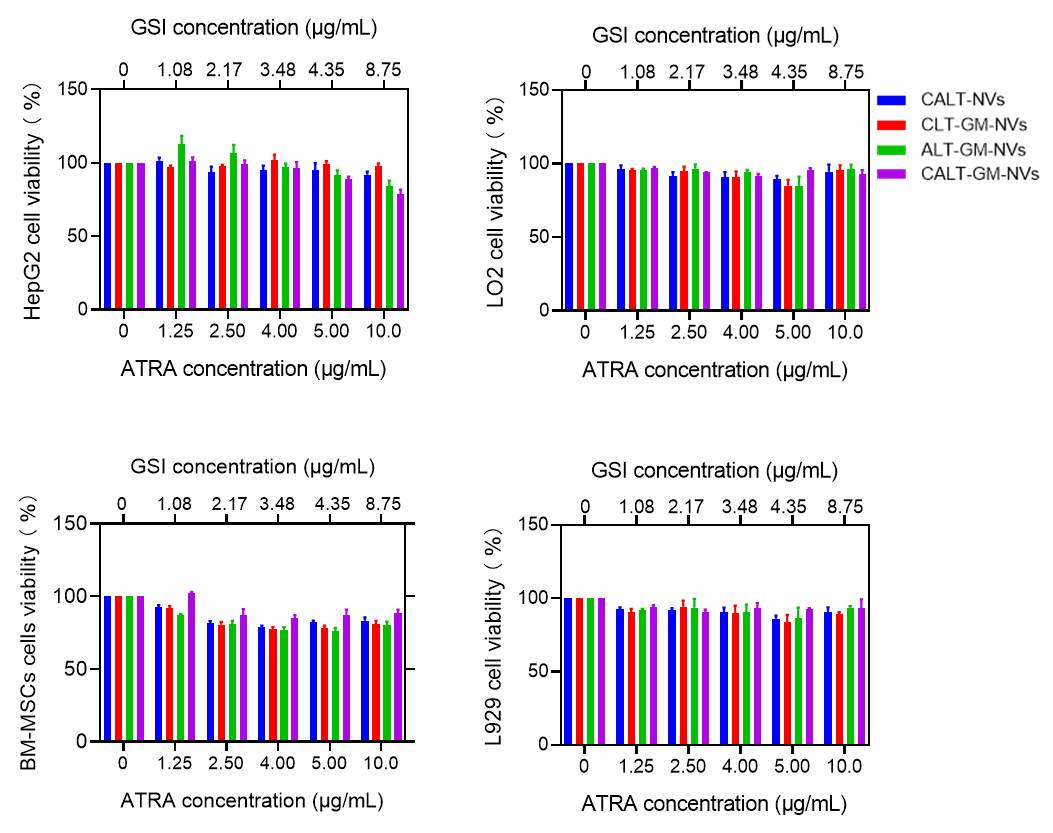
**

**Supplementary Figure 15**. Viabilities of HepG2, LO2, BM-MSCs and L929 cells treated with different concentrations of ATRA (0, 1.25, 2.5, 4.0, 5.0, and 10.0 μg/mL) and GSI (0, 1.08, 2.17, 3.48, 4.35, and 8.75 μg/mL) loaded in the biomimetic nanovesicles (*n* = 6).


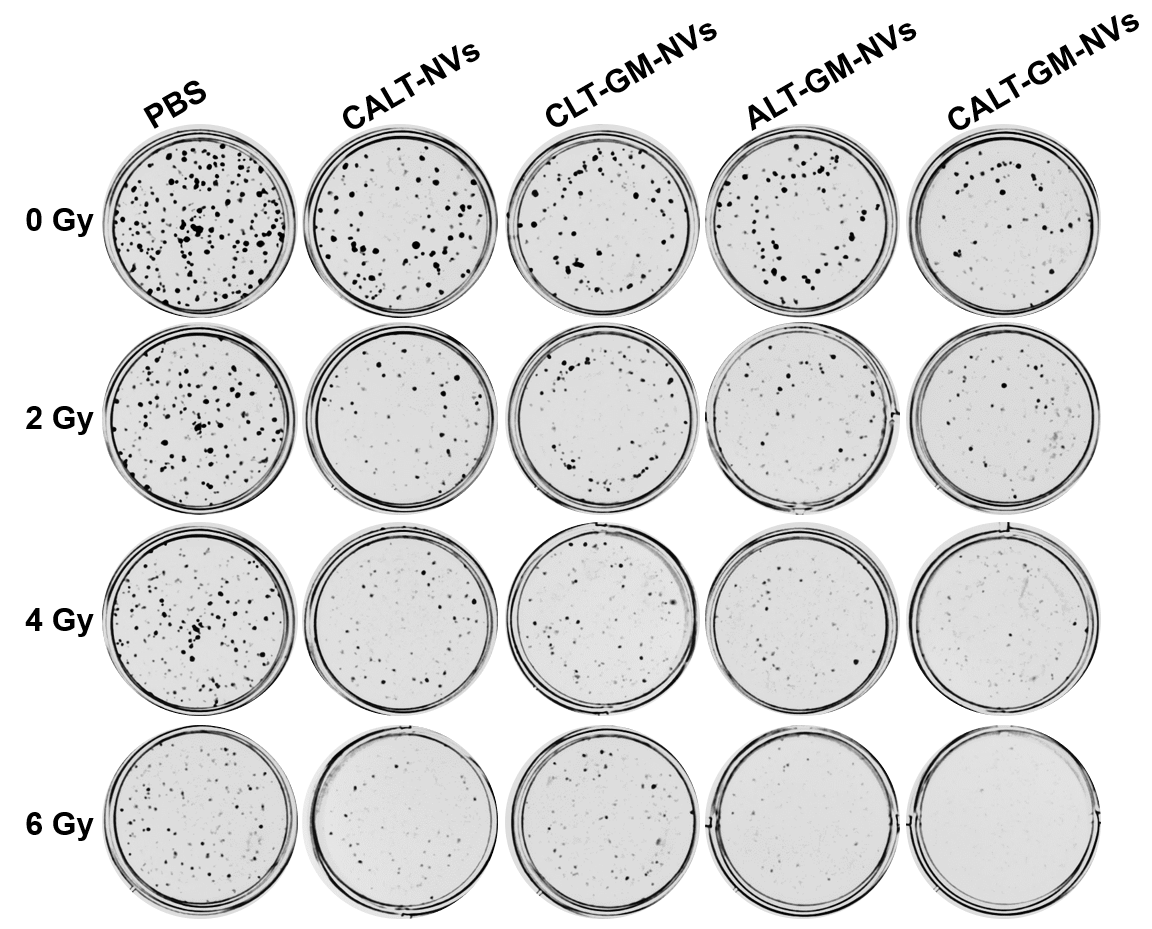


**Supplementary Figure 16.** Colony formation of HCSCs after different treatments under 0, 2, 4, or 6 Gy of radiation (corresponding to Fig. 5a).

**
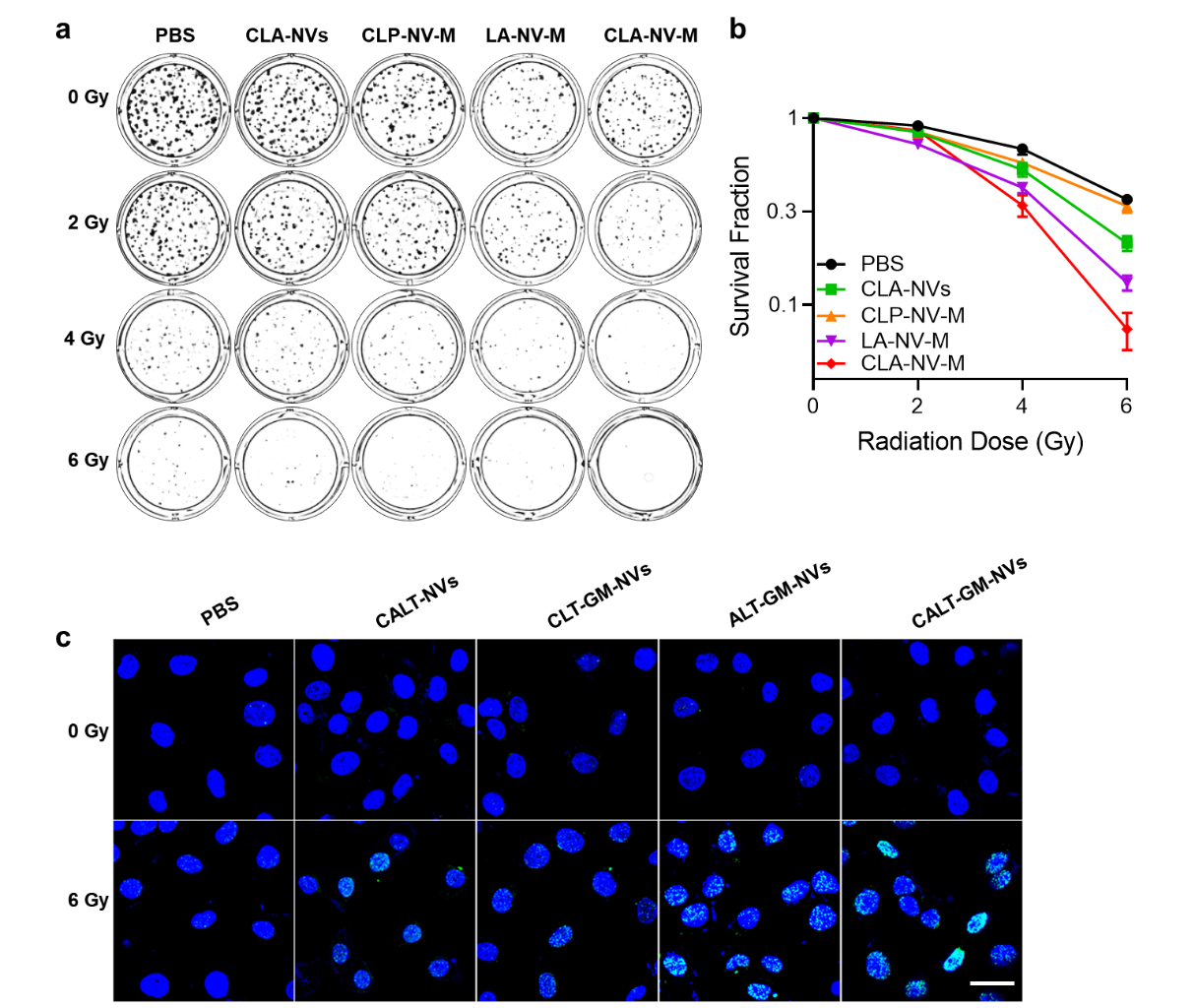
**

**Supplementary Figure 17. a** Colony formation of MCSCs after the treatment with various formulations with or without 6 Gy of radiation. **b** Clonogenic survival curves of MCSCs after the treatment with various formulations with 0, 2, 4, or 6 Gy of radiation (*n* = 6). **c** Representative images of γ-H2AX foci in MCSCs after indicated treatments (DAPI: blue).


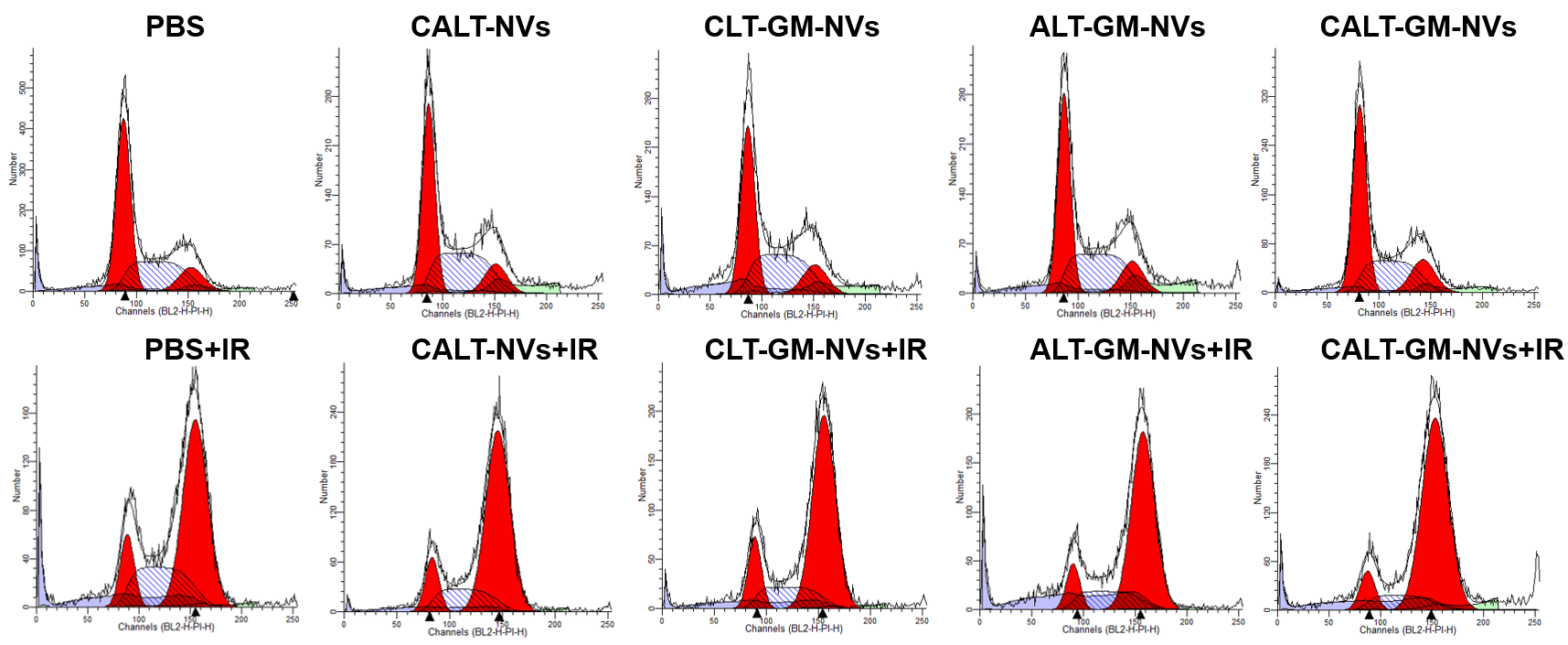


**Supplementary Figure 18.** Cell cycle distributions of HCSCs after different treatments under 0 or 6 Gy of radiation (corresponding to Fig. 5c).


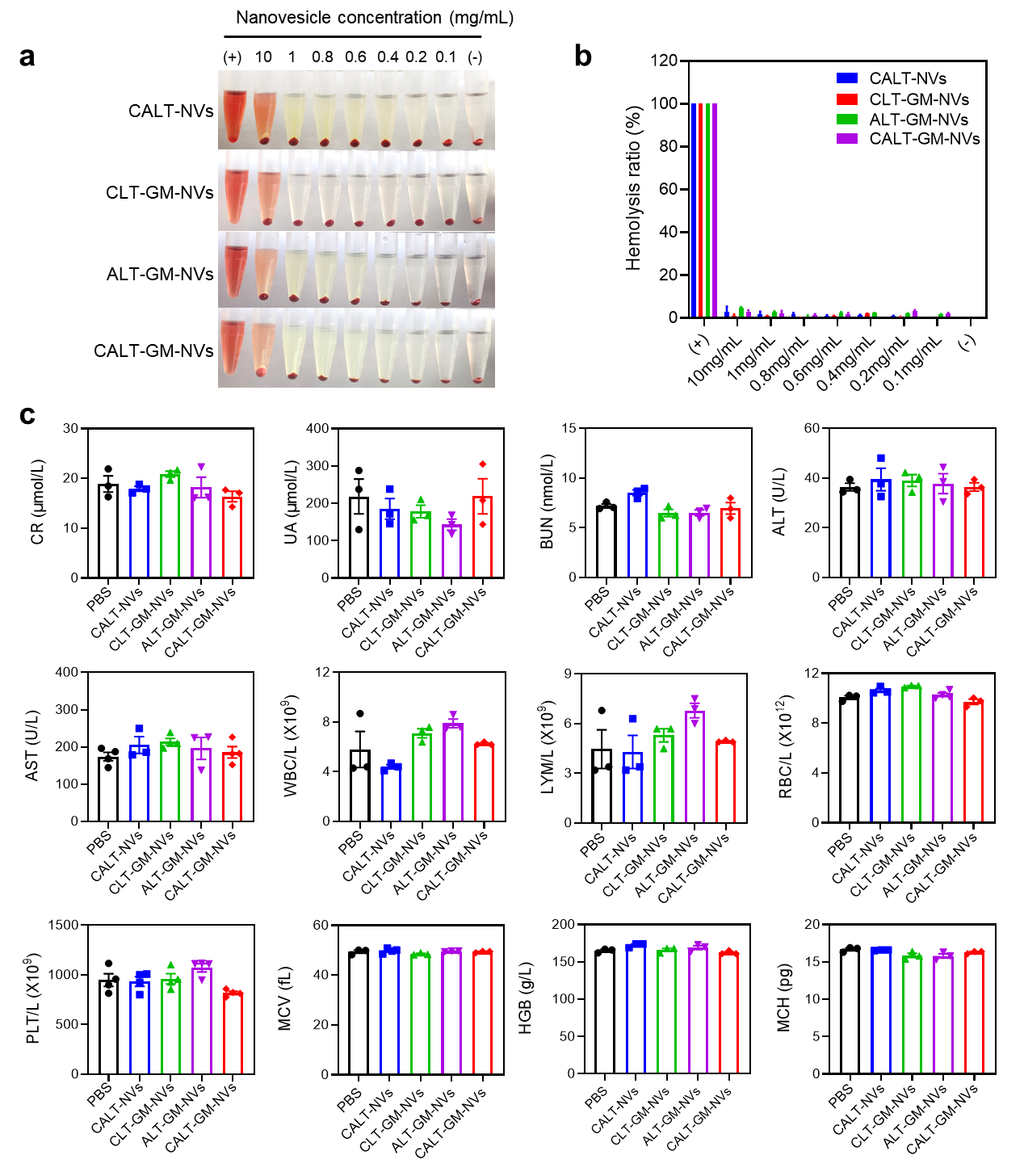


**Supplementary Figure 19. a, b** Hemolysis analysis of CALT-NVs, CLT-GM-NVs, ALT-GM-NVs and CALT-GM-NVs at pre-determined concentrations (*n* = 3). Triton X-100 and PBS were used as the positive (+) and negative (−) controls, respectively. **c** Hematology and blood biochemistry analysis of mice treated with CALT-NVs, CLT-GM-NVs, ALT-GM-NVs, or CALT-GM-NVs (*n* = 3).

**
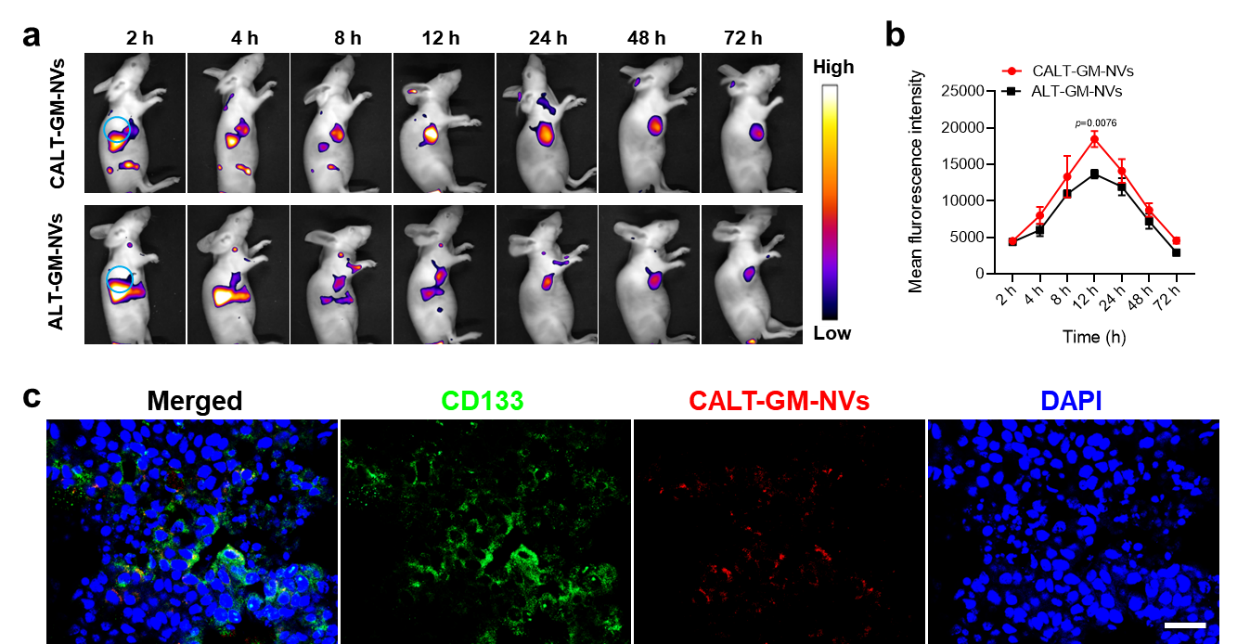
**

**Supplementary Figure 20.** *In vivo* fluorescence images (**a**) and quantification analysis (**b**) of DiR-labeled ALT-GM-NVs and CALT-GM-NVs in HepG2 liver tumor-bearing mice at indicated time points (*n* = 3). **c** CD133 (green) co-staining of tumor sections collected 12 h after intravenous injection of DiR-labeled CALT-GM-NVs (red), Scale bar: 50 μm.


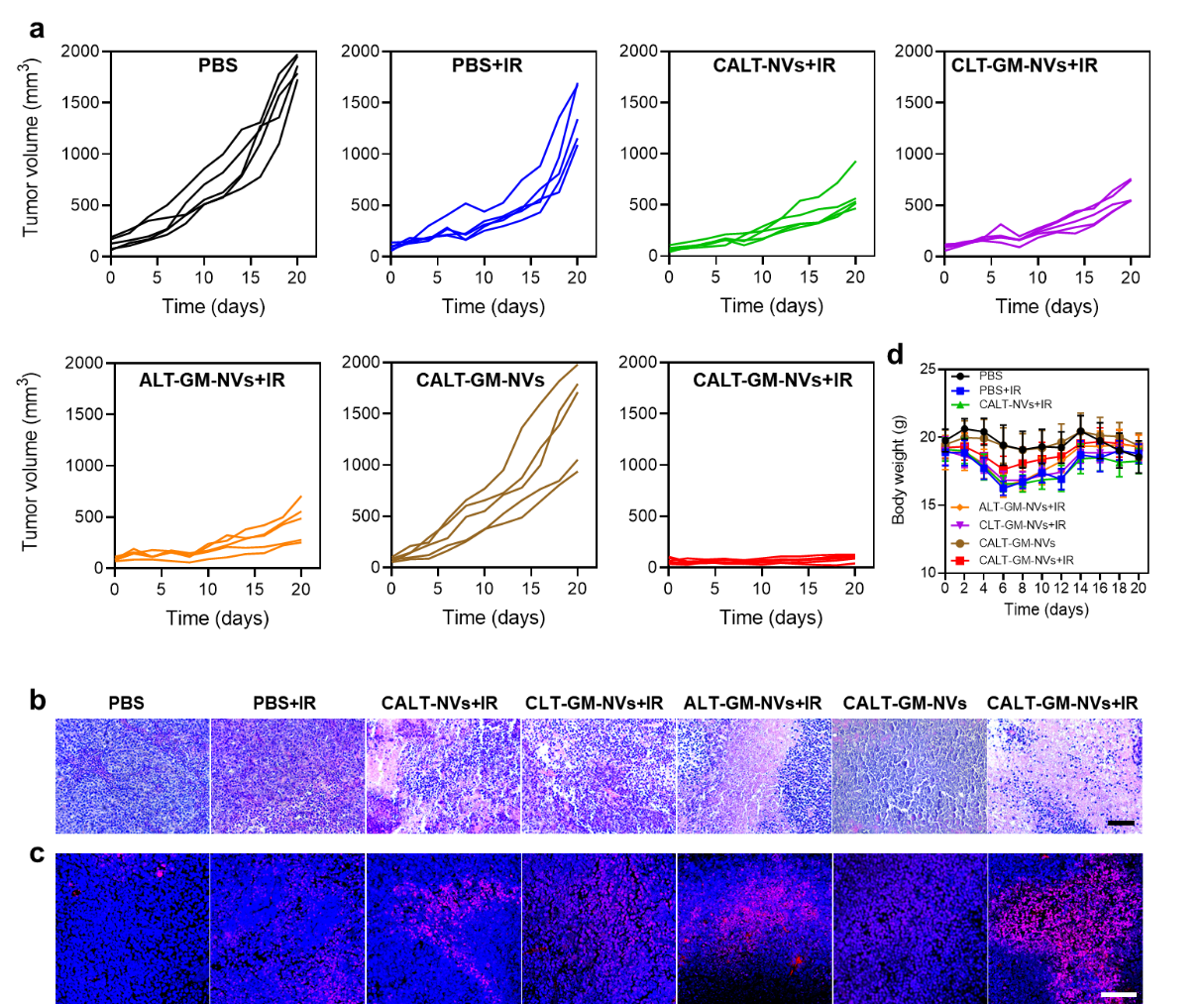


**Supplementary Figure 21.** **a** Individual tumor growth curves of HepG2 liver tumor-bearing mice with different treatments (*n* = 5). **b** H&E staining of the HepG2 tumor slices after 20 days of treatment. Scale bar: 100 μm. **c** TUNEL staining of the HepG2 tumor slices after 20 days of treatment. Scale bar: 100 μm. **d** The body weight of the HepG2 liver tumor-bearing mice with different treatments (*n* = 5).

**
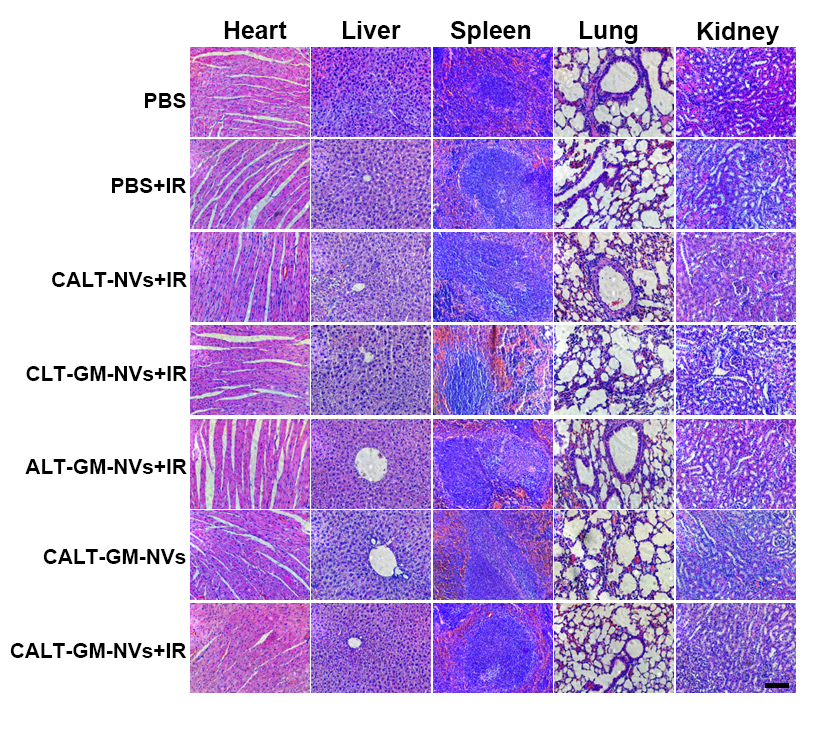
**

**Supplementary Figure 22.** H&E staining of the HepG2 major organ slices (heart, liver, spleen, lung, and kidney) after 20 days of treatment. Scale bar: 100 μm.

**Supplementary Figure 23.** Flow cytometry analysis of the percentage of CD24^+^ cells in HepG2 tumors after 22 days of treatment (*n* = 5).

**Supplementary Figure 24.** The body weight of the MHCC97H orthotopic mice after different treatments (*n* = 8).


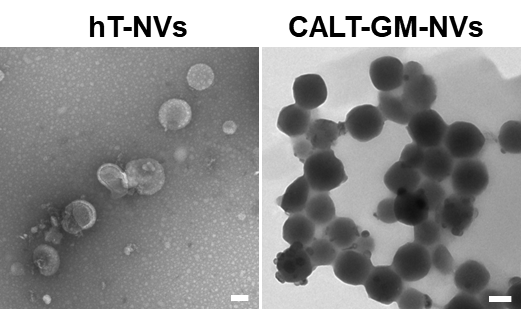


**Supplementary Figure 25.** Representative TEM images of human HCC cell membrane nanovesicles (hT-NVs) and the corresponding hT-NVs fused CALT-GM-NVs. Scale bar: 100 nm.


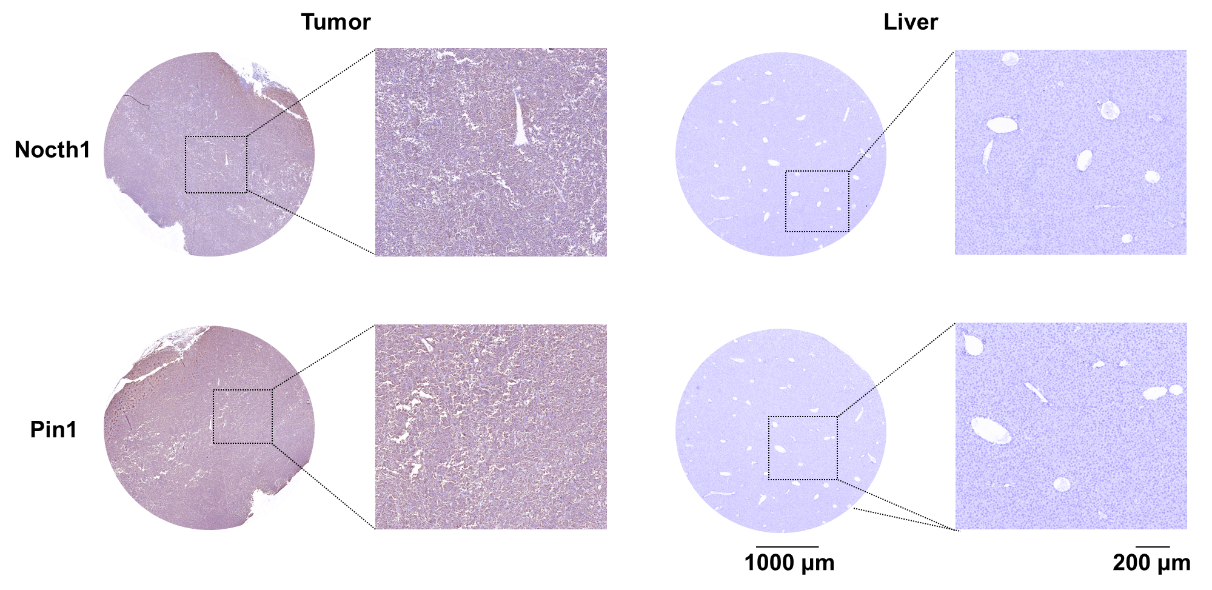


**Supplementary Figure 26.** Representative immunohistochemical staining of Pin1 and Notch1 in PDX tumor tissues and normal liver tissues.

**
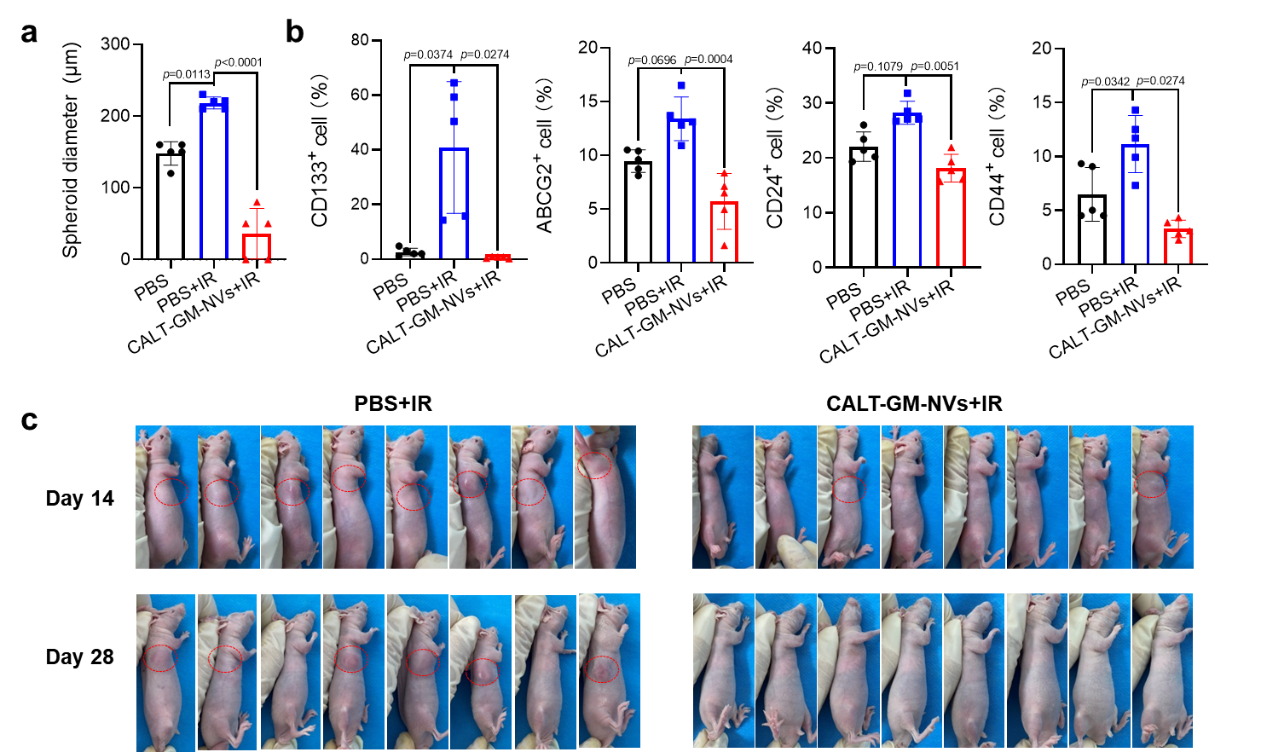
**

**Supplementary Figure 27.** **a** Quantitative analysis of tumorspheres formed by the isolated tumor cells from the PDX tumors after indicated treatments (*n* = 5). **b** Flow cytometry quantitative analysis of the percentage of CD133^+^, ABCG2^+^, CD24^+^, and CD44^+^ cells in the PDX tumors after various treatments (corresponding to Fig. 7e-h) (*n* = 5). **c** PDX tumor-bearing mice images in the PBS+IR and CALT-GM-NVs+IR groups on day 14 and 28 (*n* = 8). The red circle marks recurrent tumors.

**Table 1. Correlation between Pin1 and Notch1 expression levels and clinial parameters in 86 cases patients of HCC.**

| Variable | Total | Pin1 expression  (%) | | *p* | Notch1 expression  (%) | | *p* |
| --- | --- | --- | --- | --- | --- | --- | --- |
| Age (years) |  | High | Low | 0.724 | High | Low | 0.632 |
| <60 | 65 | 41  (75.9%) | 24  (75.0%) |  | 40  (74.0%) | 25  (78.1%) |  |
| ≥60 | 21 | 13  (24.1%) | 8  (25.0%) |  | 14  (26.0%) | 7  (21.2%) |  |
| Sex |  |  |  | 0.472 |  |  | 0.782 |
| female | 10 | 5  (9.8%) | 5  (14.3%) |  | 6  (10.9%) | 4  (12.9%) |  |
| male | 76 | 46  (90.2%) | 30  (85.7%) |  | 49  (89.1%) | 27  (87.1%) |  |
| TNM Stage |  |  |  | 0.050 |  |  | 0.004 |
| I | 55 | 26  (52.0%) | 29 (80.5%) |  | 26  (50.0%) | 29 (85.2%) |  |
| II | 28 | 21  (42.0%) | 7  (19.5) |  | 23  (46%) | 5  (14.8%) |  |
| III | 3 | 3  (6.0%) | 0  (0%) |  | 3  (6.0%) | 0  (0%) |  |
| 5-year survival | 32 | 23  (71.8%) | 9  (28.1%) | 0.096 | 22  (68.8%) | 10  (31.2%) | 0.100 |
| Disease-free survival | 48 | 32  (66.7%) | 16  (33.3%) | 0.027 | 36  (75.0%) | 12  (25.0%) | 0.004 |
| Overall survival | 44 | 35  (79.5%) | 9  (20.5%) | <0.001 | 36  (81.8%) | 8  (18.2%) | <0.001 |

**Table 2.** Half-maximal inhibitory concentration (IC_50_, μg/mL) values and combination index (CI_50_) of ATRA, GSI and ATRA combined with GSI against CSCs under 6Gy irradiation.

| ATRA alone | | | GSI alone | | | | ATRA combined with GSI | | |
| --- | --- | --- | --- | --- | --- | --- | --- | --- | --- |
| Concentration (µg/mL) | Cell viability (100%) | IC₅₀ | Concentration (µg/mL) | Cell viability (100%) | IC₅₀ | | Cell viability (100%) | IC₅₀ | CI_50_ |
| 0 | 100 | 8.10 | 0 | 100 | | 4.02 | 100 | ATRA 2.33  GSI 3.73 | 0.71 |
| 1.25 | 94.65 |  | 1.08 | 96.11 | |  | 93.60 |  |  |
| 2.5 | 82.97 |  | 2.17 | 93.84 | |  | 86.32 |  |  |
| 4 | 64.45 |  | 3.48 | 76.76 | |  | 54.43 |  |  |
| 5 | 59.02 |  | 4.35 | 78.88 | |  | 39.83 |  |  |
| 10 | 46.82 |  | 8.75 | 61.94 | |  | 22.08 |  |  |

The combination index at 50% effect (CI₅₀) was calculated using the Chou–Talalay method:

CI₅₀ = (D)₁/(Dx)₁ + (D)₂/(Dx)₂,

where (D)₁ and (D)₂ represent the concentrations of ATRA and GSI in combination that produce a 50% inhibitory effect, and (Dx)₁ and (Dx)₂ represent the concentrations of ATRA and GSI alone required to achieve the same effect. According to this method, a CI₅₀ value <1 indicates synergism, =1 indicates an additive effect, and >1 indicates antagonism.

**Table 3. Hydrodynamic diameters of GM, CALT-NVs, CLT-GM-NVs, ALT-GM-NVs, and CALT-GM-NVs (*n* = 3).**

| **Group** | **Diameter** | **Polydispersity (PDI)** |
| --- | --- | --- |
| GM | 62.8 ± 1.8 nm | 0.20 ± 0.13 |
| CALT-NVs | 94.2 ± 1.4 nm | 0.16 ± 0.04 |
| CLT-GM-NVs | 124.3 ± 4.1 nm | 0.20 ± 0.07 |
| ALT-GM-NVs | 127.9 ±1.0 nm | 0.11 ± 0.04 |
| CALT-GM-NVs | 143.0 ± 3.1 nm | 0.14 ± 0.02 |

**Table 4. The DLC and DLE of ATRA and GSI in CALT-GM-NVs (n = 3)**

|  | **DLC** | **DLE** |
| --- | --- | --- |
| ATRA | 6.71 ± 0.19% | 42.40 ± 2.13% |
| GSI | 5.86 ± 0.10% | 41.04 ± 0.70% |

Table 5. Primers used in the qRT-PCR assay (human).

| **Transcription** | **Primer sequences** |
| --- | --- |
| *Notch1* | Forward: CTGAAGAACGGGGCTAACAA  Reverse: CAGGTTGTACTCGTCCAGCA |
| *Pin1* | Forward: CATCACTAACGCCAGCCAGT  Reverse: TCAAATGGCTTCTGCATCTG |
| *HES-1* | Forward: GAGAAAAGACGAAGAGCA  Reverse: TGTGCTCAGCGCAGCCGT |
| *HEY-1* | Forward: GAGTGCGGACGAGAATGGAA  Reverse: TCGTCGGCGCTTCTCAATTA |
| *KLF-4* | Forward: ATTACGCGGGCTGCGGCAAAA  Reverse: TTTTTGGCACTGGAACGGGCGG |
| *BIRC-5* | Forward: CTTAGAGGGACAAGTGGCGTTC  Reverse: CGCTGAGCCAGTCAGTGTAG |
| *Nanog* | Forward: GTCCCAAAGGCAAACAACCC  Reverse: GCTGGGTGGAAGAGAACACA |
| *Oct4* | Forward: CTTGAATCCCGAATGGAAAGGG  Reverse: GTGTATATCCCAGGGTGATCCTC |
| *Sox2* | Forward: GCCCTGCAGTACAACTCCAT  Reverse: GACTTGACCACCGAACCCAT |
| *β-actin* | Forward: CTTAGAGGGACAAGTGGCGTTC  Reverse: CGCTGAGCCAGTCAGTGTAG |
|  |  |
